# Supplementary material for: Structural mapping of PEAK pseudokinase interactions identifies 14-3-3 as a molecular switch for PEAK3 signaling
Source: Nat Commun. 2023 Jun 19;14:3542. doi: 10.1038/s41467-023-38869-9 (PMC10279719; doi:10.1038/s41467-023-38869-9)
Supplement: Supplementary file 1 — Supplementary Information [file 41467_2023_38869_MOESM1_ESM.pdf]

Supplementary Information

Structural mapping of PEAK pseudokinase interactions identifies 14-3-3 as a molecular switch for PEAK3 signaling.

Michael J. Roy[1,2,#], Minglyanna G. Surudoi[1,2], Ashleigh Kropp[1,2], Jianmei Hou[3,4,] Weiwen Dai[1,2], Joshua M. Hardy[1,2], Lung-Yu Liang[1,2], Thomas R. Cotton[1,2], Bernhard C. Lechtenberg[1,2], Toby A. Dite[1,2], Xiuquan Ma[3,4], Roger J. Daly[3,4], Onisha Patel[1,2*] and Isabelle S. Lucet[1,2,*,#]

[1.] The Walter and Eliza Hall Institute of Medical Research, Parkville, VIC 3052, Australia.

[2.] Department of Medical Biology, University of Melbourne, Parkville, VIC 3052, Australia.

[3.] Cancer Program, Biomedicine Discovery Institute, Monash University, Melbourne, VIC 3800, Australia.

[4.] Department of Biochemistry and Molecular Biology, Monash University, Melbourne, VIC 3800, Australia.

[*] These authors jointly supervised this work.

[#]Correspondence: roy@wehi.edu.au, lucet.i@wehi.edu.au

Contents

Supplementary Table 1. Purification buffers for adaptor/scaffold proteins.

Supplementary Table 2. Purification buffers for PEAK proteins.

Supplementary Table 3. Purification buffers for kinase domains for *in vitro* phosphorylation reactions.

Supplementary Fig. 1. Multiple Sequence Alignment (MSA) of PEAK vertebrate orthologues.

Supplementary Fig. 2. Interaction of PEAK SH2 peptides with Grb2 and CrkII.

Supplementary Fig. 3. Biophysical analysis of PEAK/CrkII interaction.

Supplementary Fig. 4. PEAK3:14-3-3 interaction.

Supplementary Fig. 5. Structural and biophysical characterisation of PEAK phosphopeptides/14-3-3 interaction.

Supplementary Fig. 6. ITC competition binding studies.

Supplementary Fig. 7. Proposed model for PEAK protein interactions.

Supplementary Table 1. Purification buffers for adaptor/scaffold proteins.

**Buffers**	**14-3-3 isoforms**	**CrkII^{FL}**<br>**CrkII$^{\Delta CSH3}$**<br>**CrkII^{NSH3}**<br>**CrkII^{SH2}**	**Grb2^{FL}**<br>**Grb2^{SH2}**
**Lysis**	20 mM Tris pH 7.5<br>500 mM NaCl<br>5 mM DTT<br>5 mM imidazole<br>10% (v/v) glycerol<br>0.1% (w/v) Thesit<br>2 mM EDTA	20 mM Tris pH 7.5<br>500 mM NaCl<br>5 mM DTT<br>5 mM imidazole<br>10% (v/v) glycerol<br>0.1% (w/v) Thesit	20 mM Tris pH 7.5<br>500 mM NaCl<br>5 mM DTT<br>5 mM imidazole<br>10% (v/v) glycerol<br>0.1% (w/v) Thesit
**Ni-Affinity Wash**	20 mM Tris pH 7.5<br>500 mM NaCl<br>5 mM DTT<br>5 mM imidazole<br>10% (v/v) glycerol<br>2 mM EDTA	20 mM Tris pH 7.5<br>500 mM NaCl<br>5 mM DTT<br>5 mM imidazole<br>10% (v/v) glycerol	20 mM Tris pH 7.5<br>500 mM NaCl<br>5 mM DTT<br>5 mM imidazole<br>10% (v/v) glycerol
**Ni-Affinity Elution**	Ni-Affinity Wash Buffer<br>250 mM imidazole	Ni-Affinity Wash Buffer<br>250 mM imidazole	Ni-Affinity Wash Buffer<br>250 mM imidazole
**SEC**	20 mM HEPES pH 7.5<br>150 mM NaCl<br>0.5 mM TCEP	20 mM Tris pH 7.5<br>200 mM NaCl<br>0.5 mM TCEP<br>5% (v/v) glycerol	20 mM Tris pH 7.5<br>200 mM NaCl<br>0.5 mM TCEP<br>5% (v/v) glycerol
**IEX Buffer A**	20 mM HEPES pH 7.5<br>0.5 mM TCEP	20 mM HEPES pH 7.5<br>0.5 mM TCEP	20 mM HEPES pH 7.5<br>0.5 mM TCEP<br>5% (v/v) glycerol
**IEX Buffer B**	20 mM HEPES pH 7.5<br>1 M NaCl<br>0.5 mM TCEP	20 mM HEPES pH 7.5<br>1 M NaCl<br>0.5 mM TCEP	20 mM HEPES pH 7.5<br>1 M NaCl<br>0.5 mM TCEP<br>5% (v/v) glycerol

Supplementary Table 2. Purification buffers for PEAK proteins.

Buffers	PEAK1^{IDR1}	PEAK2^{IDR1}	PEAK3^{FL}
**Lysis**	20 mM Tris pH 8.0<br>500 mM NaCl<br>5 mM DTT<br>10 mM imidazole<br>10% (v/v) glycerol<br>0.1% (w/v) Thesit<br>2 mM EDTA	20 mM Tris pH 8.5<br>500 mM NaCl<br>10 mM DTT<br>5 mM imidazole<br>10% (v/v) glycerol<br>0.1% (w/v) Thesit<br>2 mM EDTA	20 mM Tris pH 7.5<br>500mM NaCl<br>10 mM DTT<br>20 mM imidazole<br>10% (v/v) glycerol<br>0.1% (w/v) Thesit
**Ni-Affinity Wash**	20 mM Tris pH 8<br>500 mM NaCl<br>5 mM DTT<br>10 mM imidazole<br>10% (v/v) glycerol<br>2 mM EDTA	20 mM Tris pH 8.5<br>500 mM NaCl<br>10 mM DTT<br>5 mM imidazole<br>10% (v/v) glycerol<br>2 mM EDTA	20 mM Tris pH 7.5<br>500 mM NaCl<br>10 mM DTT<br>20 mM imidazole,<br>10% (v/v) glycerol
**Ni-Affinity Elution**	Ni-Affinity Wash Buffer<br>250 mM imidazole	Ni-Affinity Wash Buffer<br>250 mM imidazole	Ni-Affinity Wash Buffer<br>250 mM imidazole
**SEC**	20 mM Tris pH 8.0<br>200 mM NaCl<br>0.5 mM TCEP<br>5% (v/v) glycerol	20 mM Tris pH 8.5<br>200 mM NaCl<br>1 mM TCEP<br>5% (v/v) glycerol	20 mM Tris pH 7.5<br>250 mM NaCl<br>1 mM TCEP
**IEX Buffer A**	20 mM Tris pH 8.0<br>1 mM TCEP<br>5% (v/v) glycerol	N/A	20 mM Tris pH 7.5<br>1 mM TCEP
**IEX Buffer B**	20 mM Tris pH 8.0<br>1 M NaCl<br>1 mM TCEP<br>5% (v/v) glycerol	N/A	20 mM Tris pH 7.5<br>1 M NaCl<br>1 mM TCEP

Supplementary Table 3. Purification buffers for kinase domains for *in vitro* phosphorylation reactions.

Buffers	Src	Abl
**Lysis**	50 mM Tris pH 8.0<br>500 mM NaCl<br>1 mM DTT<br>10 mM imidazole<br>5% (v/v) glycerol<br>0.1% (w/v) Thesit	20 mM Tris pH 7.5<br>500 mM NaCl<br>5 mM DTT<br>5 mM imidazole<br>10% (v/v) glycerol<br>0.1% (w/v) Thesit
**Ni-Affinity Wash**	50 mM Tris pH 7.5<br>500 mM NaCl<br>1 mM DTT<br>25 mM imidazole<br>5% (v/v) glycerol	20 mM Tris pH 7.5<br>500 mM NaCl<br>5 mM DTT<br>5 mM imidazole<br>10% (v/v) glycerol
**Ni-Affinity Elution**	Ni-Affinity Wash Buffer<br>250 mM imidazole	Ni-Affinity Wash Buffer<br>250 mM imidazole
**SEC**	20 mM Tris pH 8.0<br>150 mM NaCl<br>1 mM DTT	20 mM Tris pH 7.5<br>150 mM NaCl<br>5% (v/v) glycerol<br>0.5 mM TCEP
**IEX Buffer A**	20 mM Tris pH 8.0<br>1 mM TCEP	N/A
**IEX Buffer B**	20 mM Tris pH 8.0<br>1 M NaCl<br>1 mM TCEP	N/A

Supplementary Figures.

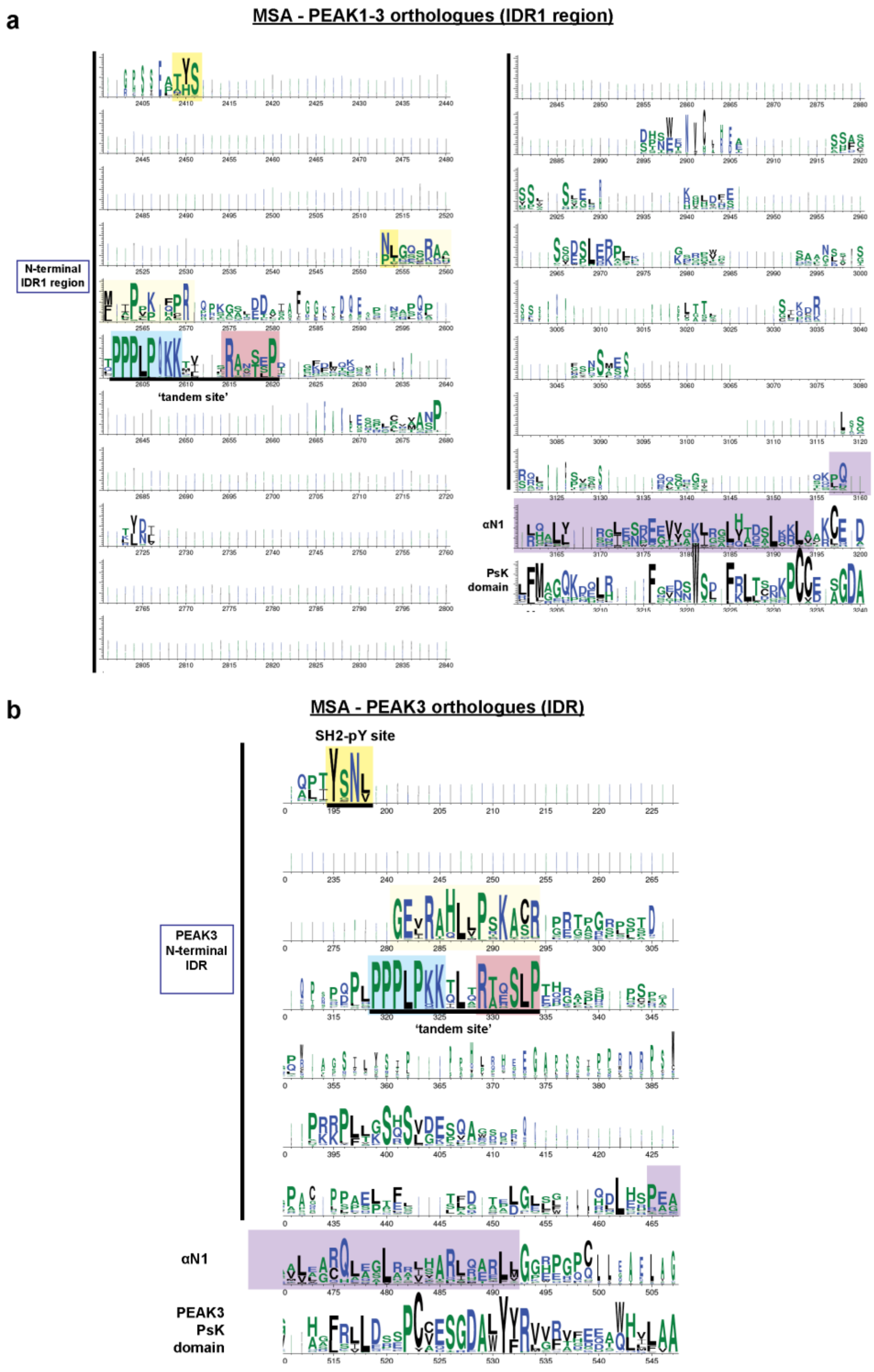

Supplementary Fig. 1. Multiple Sequence Alignment (MSA) of PEAK vertebrate orthologues. **a** MSA/Web Logo of conservation across PEAK3/PEAK1/PEAK2 vertebrate orthologs (N-IDR1 region). **b** Full MSA/Web Logo of PEAK3 orthologs (N-terminal IDR1 region). Regions of interest for this study are highlighted, in particular the conserved SH2 pY binding site present in PEAK3/PEAK1 (TYSNL motif) and the 'tandem site' (encompassing proline rich motif known to bind CrkII^{NSH3} and putative 14-3-3 binding motif).

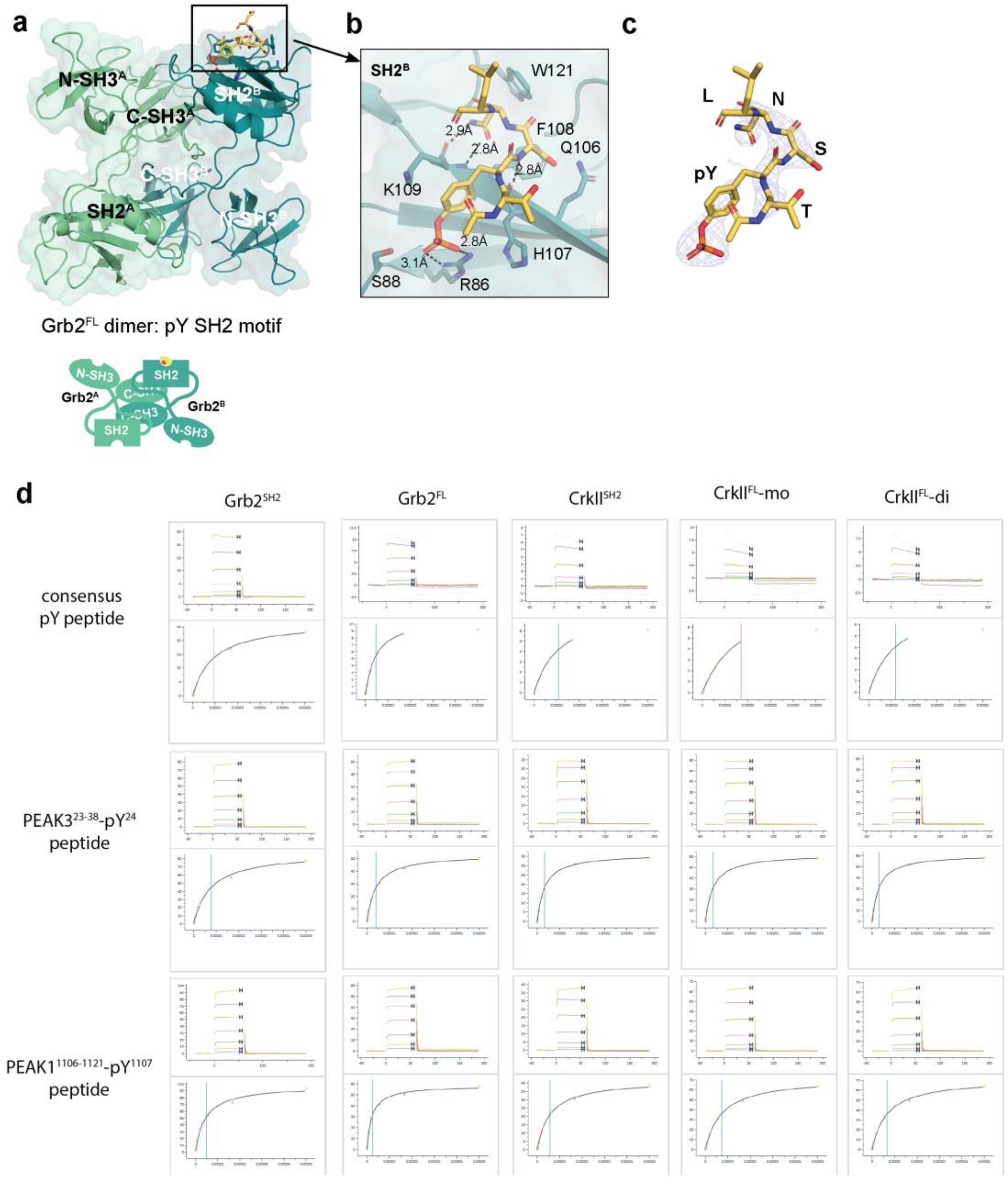

Supplementary Fig. 2. Interaction of PEAK SH2 peptides with Grb2 and CrkII. **a** Ribbon representation of the structure of Grb2^{FL}:PEAK SH2-pY peptide showing dimer arrangement (chain A in light green and chain B in dark green) with cartoon illustrating domain organization. **b** Zoom in highlighting peptide groove and the PEAK phosphopeptide (yellow) adopting a characteristic β-turn forming three important hydrogen bonds to the Grb2 backbone with residues Arg 86, His 107 and Lys109. Peptide and interacting residues of Grb2 displayed in a stick representation. **c** Unbiased Fo-Fc omit map (grey mesh, contoured at 3.0 σ) showing peptide density prior to modelling and final modelled PEAK3/PEAK1 phosphopeptide (T**pY**SNLGQ, yellow sticks, underlined residues modelled). **d** Representative SPR sensorgrams and steady state fitting for binding of the consensus pY-SH2 motif and PEAK3$^{23-38}$-pY^{24} and PEAK1$^{1106-1121}$-pY^{1107} peptides to recombinant full-length Grb2/CrkII forms and corresponding SH2 domains (See Supplementary Data 1 for full SPR data).

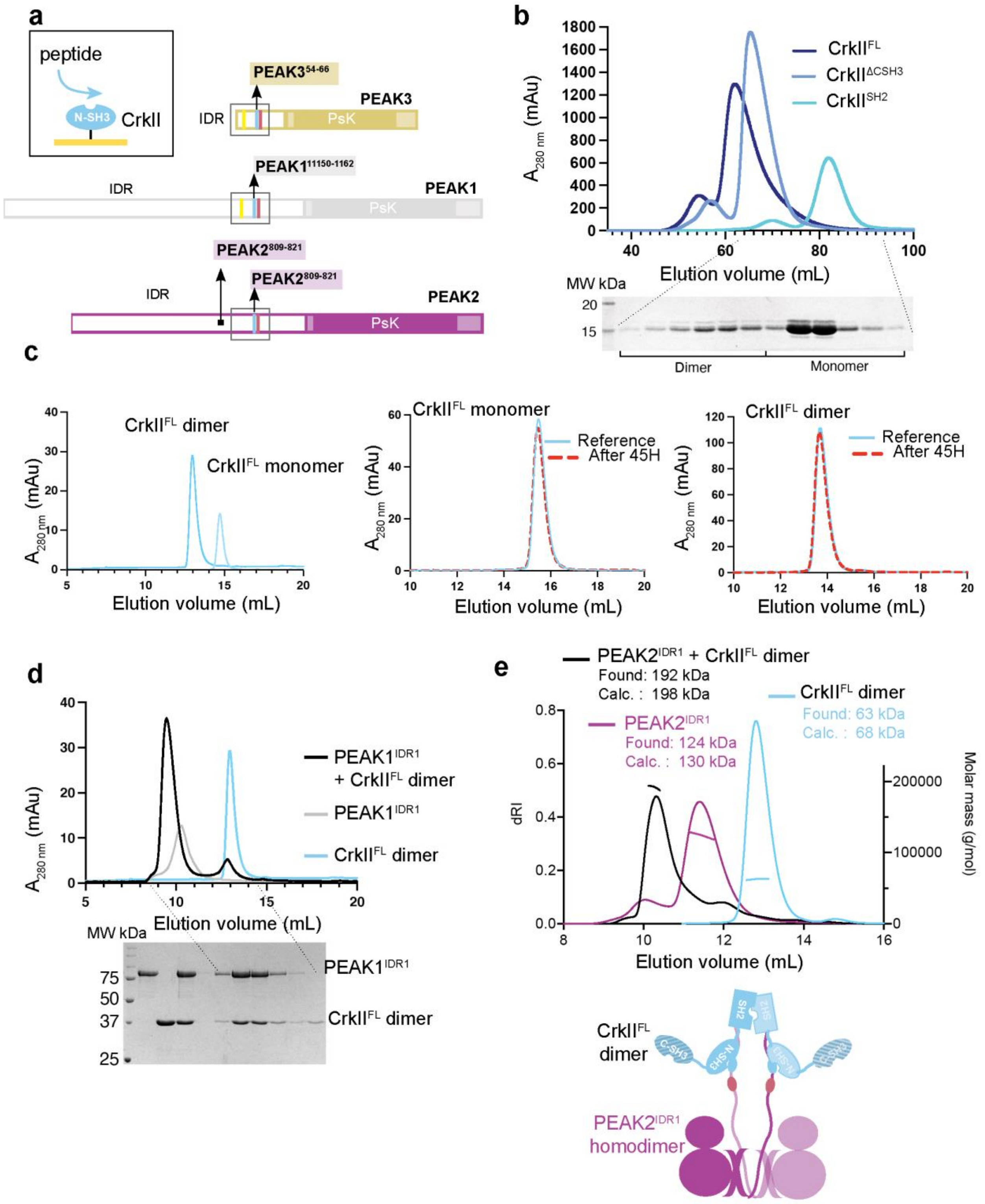

Supplementary Fig. 3. Biophysical analysis of PEAK/CrkII interaction. a Schematic highlighting synthetic peptides generated for PEAK1, PEAK2 or PEAK3 encompassing Proline Rich Motifs (PRMs) predicted to bind CrkII^{NSH3} domain. **b** Size exclusion chromatography (SEC) profile (S75 16/600) of CrkII^{FL} and truncated CrkII$^{\Delta CSH3}$ and CrkII^{SH2} constructs showing a similar proportion of dimer, indicating that the SH2 domain mediates dimerization (n = 3, independent experiments). **c** Overlaid SEC profiles (S200 10/300) of individually purified CrkII^{FL} dimer and CrkII^{FL} monomer

(left) and of CrkII^{FL} monomer (middle) and CrkII^{FL} dimer (right) after 45 hours incubation at 4°C, demonstrating their stability. **d** Incubation of PEAK1^{IDR1} with CrkII^{FL} dimer (1:1.3 molar ratio) results in formation of a stable complex, as analysed by SEC confirmed by SDS-PAGE analysis of eluted fractions (n = 3, independent experiments). **e** SEC-MALS analysis of PEAK2^{IDR1} dimer:CrkII^{FL} dimer complex and PEAK2^{IDR1} dimer alone (n = 2, independent experiments). Absorbance was measured at a wavelength of 280 nm. Theoretical and observed molecular mass values are indicated. A diagram of the PEAK2^{IDR1} dimer:CrkII^{FL} dimer complex is shown.

a

Protein (from *Sf.* 21 insect cells)	14-3-3 motif	14-3-3 motif phosphorylated (LC MS/MS)
**PEAK3**[FL]	RTQ**S**LP	Y
**PEAK1**[IDR1]	RANTEP	N
**PEAK2**[IDR1]	RAA**S**SP	Y

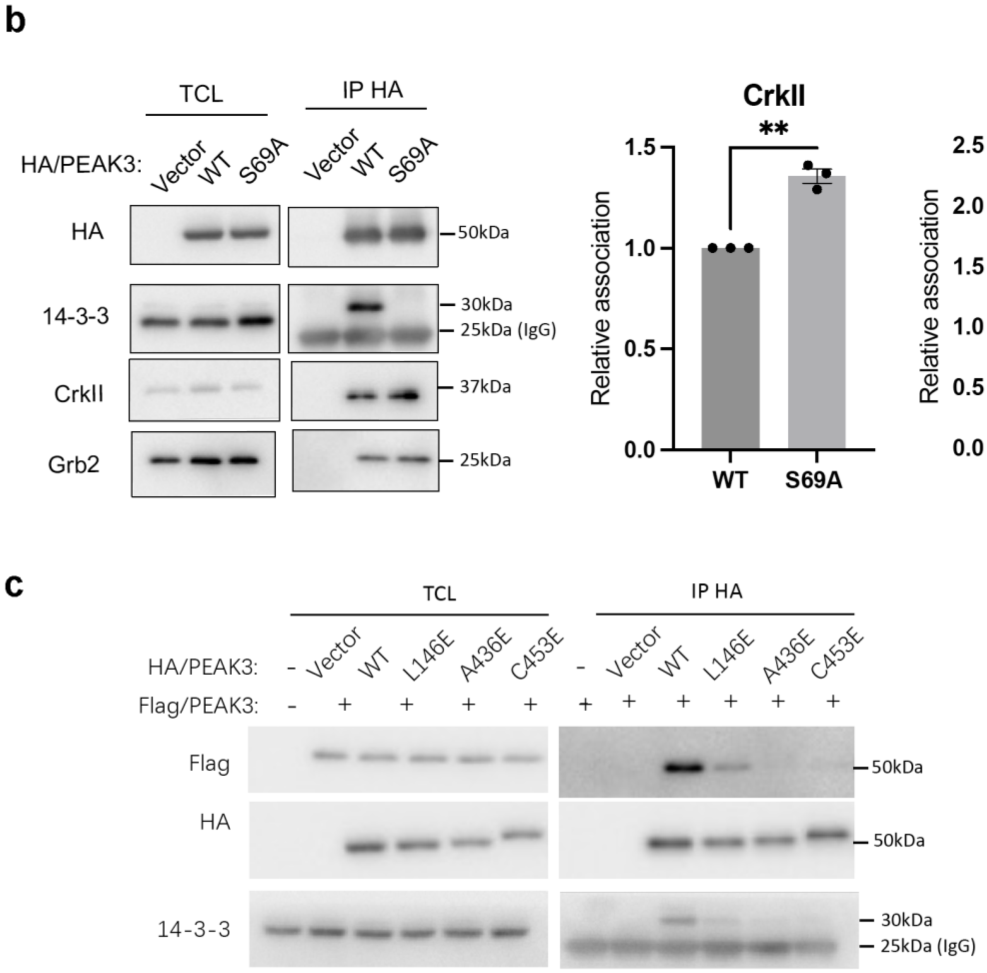

Supplementary Fig. 4. PEAK3:14-3-3 interaction. a Table summarising phosphorylation state at the tandem site 14-3-3 motif as identified by LC MS/MS for recombinant PEAK proteins purified from insect cells. Mass-directed tryptic proteomics proteomic analysis identifying phosphorylation sites for recombinantly purified PEAK proteins, as well as identification of 14-3-3 isoforms in the purified PEAK3^{FL}:14-3-3 complex purified from insect cells are both available as a Source Data File. **b** Western blot analysis for immunoprecipitation of HA-tagged PEAK3^{FL} WT and PEAK3^{FL} S69A expressed in HEK293 cells and probed using anti-HA, anti-14-3-3 anti-CrkII and anti-Grb2; showing that the S69 site is required for 14-3-3 co-immunoprecipitation with PEAK3^{FL} from cells and that mutation of this critical 14-3-3 site (PEAK3^{FL} S69A mutant) is concomitant with a modest increase in the observed level of CrkII and Grb2 bound to PEAK3^{FL}. Densitometry data quantifying the mean level relative association of CrkII to HA-PEAK3^{FL} S69A relative to HA- PEAK3^{FL} WT ($p = 0.0073$),

and of Grb2 to HA-PEAK3^{FL} S69A relative to HA- PEAK3^{FL} WT (p = 0.0378). Western blot and densitometry data represent three independent repeats; uncertainties are S.E.M; ** p<0.01, by ratio paired t-test. Repeats shown in Source Data. **c** PEAK3 mutants (L146E, A436E, C453E) that disrupt dimerization reduce 14-3-3 interaction with PEAK3. Flag-tagged PEAK3 WT and HA-tagged PEAK3 WT or dimerization mutants were co-expressed in HEK293 cells. Anti-HA IPs were prepared from cell lysates and Western blotted as indicated. (n=3 biologically independent repeats). Repeats shown in Source Data. Source data are provided as a source data file.

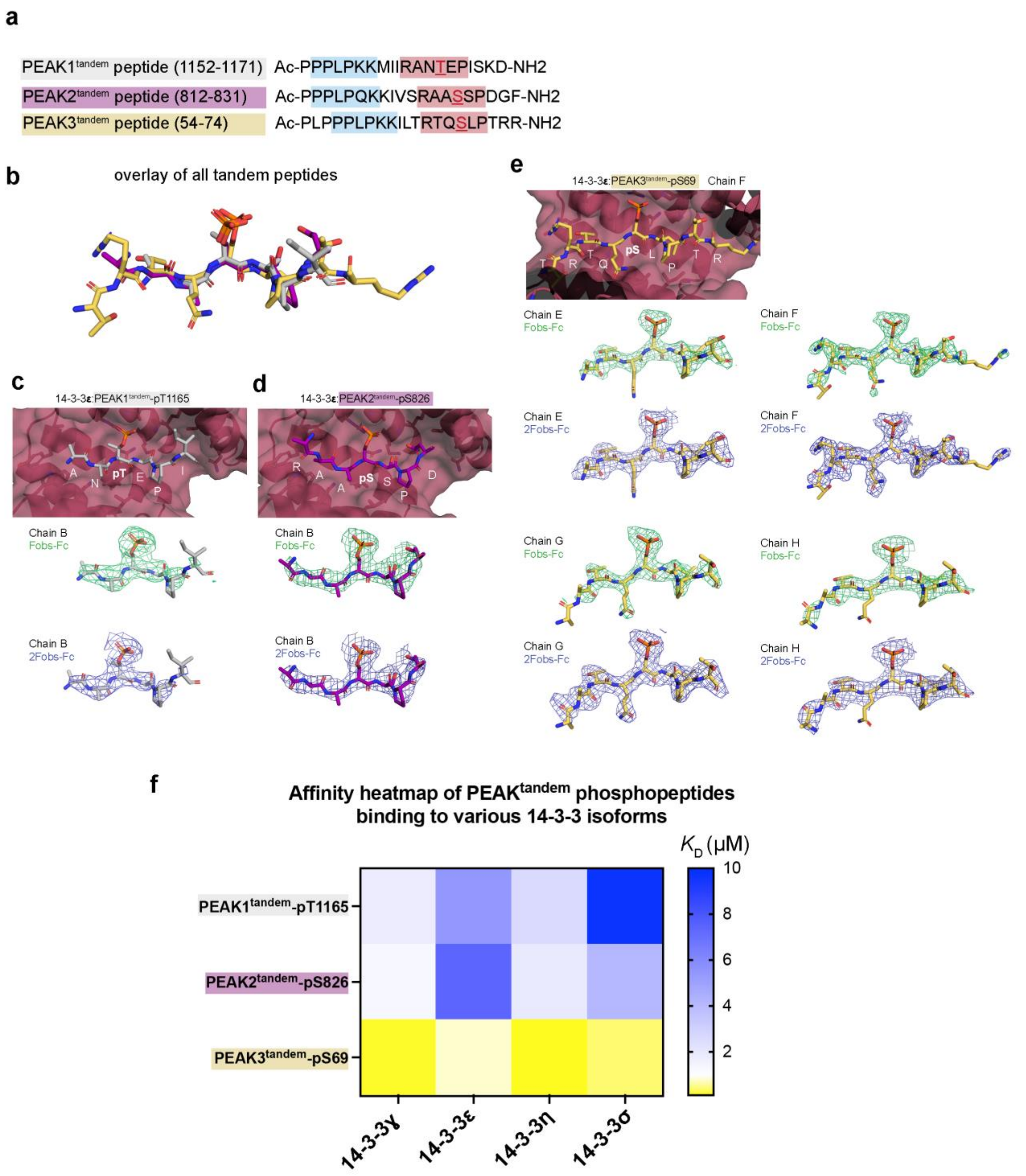

Supplementary Fig. 5. Structural and biophysical characterisation of PEAK phosphopeptides/14-3-3 interaction. **a** Primary sequence of PEAK tandem peptides; phosphorylated or non-phosphorylated at the underlined residue (red). **b** Comparison of crystal structures of PEAK tandem phosphopeptides with 14-3-3ε, showing overlay of all three PEAK peptides. **c** PEAK1^{tandem}-pT1165 (Chain B, grey sticks). **d** PEAK2^{tandem}-pS826 (Chain B, purple sticks). **e** PEAK3^{tandem}-pS69 (Chain F, yellow sticks) and peptides bound to the groove of 14-3-3ε (maroon surface/cartoon) and demonstrating phospho-recognition by 14-3-3. For each 14-3-3-bound

phosphopeptide (unique chain) in each structure, the corresponding electron density map for the modelled peptide in the final structure is shown (green, simulated anneal omit map with peptide removed, Fo-Fc, contoured at 3.0σ, carve radius 2.0 Å; blue, refined electron density map, 2Fo-Fc, contoured at 1.0σ, carve radius 2.0 Å). **f** Heatmap of SPR steady state affinity values (K_D) of human 14-3-3 isoforms for phosphorylated PEAK tandem peptides (See Supplementary Data 4 for tabulated SPR data).

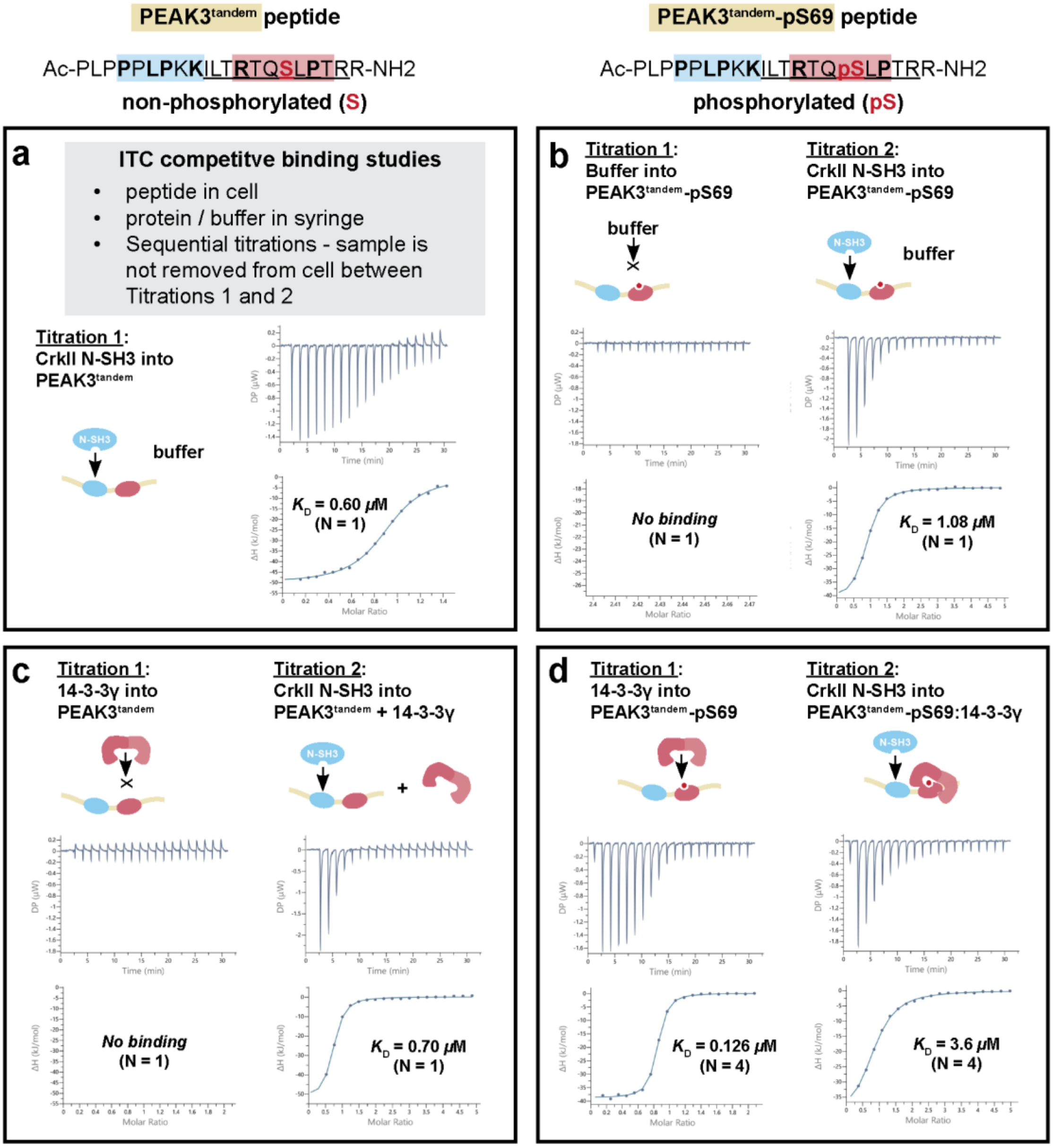

Supplementary Fig. 6. ITC competition binding studies. Sequential ITC binding studies using: **a, c,** PEAK3^{tandem}-S69 (non-phosphorylated) and **b, d**, PEAK3^{tandem}-pS69 (phosphorylated) peptides and purified 14-3-3ɣ and CrkII^{NSH3} domain. Corresponding ITC derived affinity values (K_D) (mean value, N = 1 (**a**) or N = 4 (**b-d**) independent experiments (N). (See Supplementary Data 5 for tabulated ITC data).

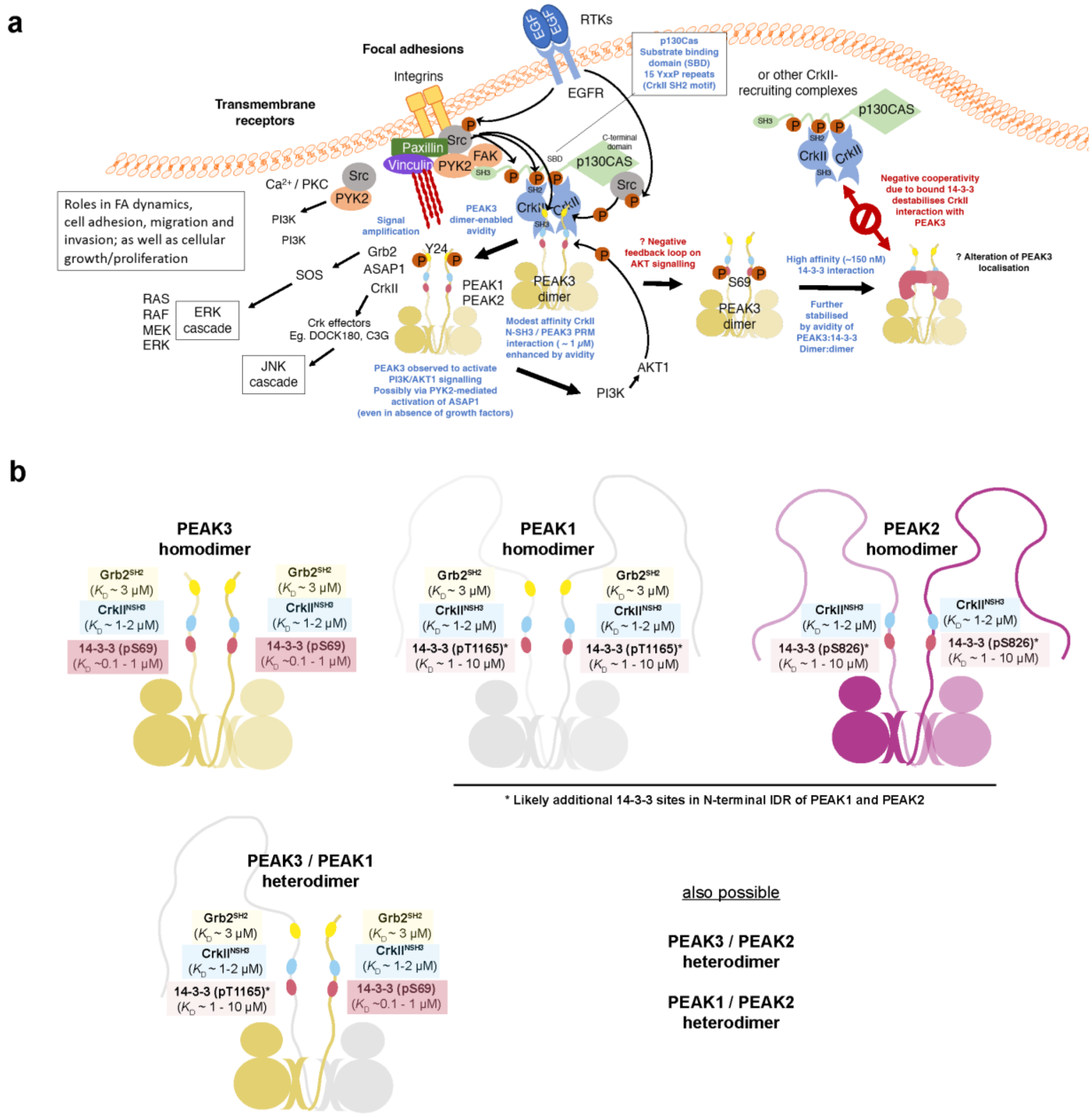

Supplementary Fig. 7. Proposed model for PEAK protein interactions. a Schematic of PEAK signaling pathways at focal adhesions. **b** Schematic summarizing the approximate binding affinity (K_D for 1:1 interaction) of Grb2, CrkII and 14-3-3 towards PEAK1/PEAK2/PEAK3 dimers at N-terminal IDR motifs examined in this study, highlighting PEAK homodimer and heterodimer configurations and diverse interaction outputs.
