# Supplementary material for: Structural mapping of PEAK pseudokinase interactions identifies 14-3-3 as a molecular switch for PEAK3 signaling
Source: Nat Commun. 2023 Jun 19;14:3542. doi: 10.1038/s41467-023-38869-9 (PMC10279719; doi:10.1038/s41467-023-38869-9)
Supplement: Supplementary file 3 — Description of Additional Supplementary Files [file 41467_2023_38869_MOESM3_ESM.pdf]

Description of Additional Supplementary Files

Supplementary Data 1	Supplementary Data Table 1: SPR-determined steady state binding affinity of PEAK pY SH2 motifs with CrkII and Grb2 (full length and SH2 domains)<br>SPR sensorgrams and fitted steady state data
Supplementary Data 2	Supplementary Data Table 2: SPR-determined steady state binding affinity of PEAK proline rich motif (PRM) peptides with CrkII N-SH3<br>Representative SPR sensorgrams and fitted steady state data for PEAK proline rich motif (PRM) peptides binding to immobilised CrkII N-SH3
Supplementary Data 3	Supplementary Data Table 3: SPR-determined steady state binding affinity of PEAK tandem peptides with CrkII N-SH3<br>Representative SPR sensorgrams and fitted steady state data for PEAK proline rich motif (PRM) peptides binding to immobilised CrkII N-SH3
Supplementary Data 4	Supplementary Data Table 4: SPR-determined steady state binding affinity of PEAK tandem peptides with 14-3-3 isoforms<br>Representative SPR sensorgrams and fitted steady state data for PEAK tandem peptides binding to immobilised 14-3-3 isoforms
Supplementary Data 5	Supplementary Data Table 5A: Summary of individual ITC binary and ternary experiments for PEAK3 tandem peptides with 14-3-3 and CrkII N-SH3<br>Supplementary Data Table 5B: Summary of mean data for ITC binary and ternary experiments for PEAK3 tandem peptides with 14-3-3 and CrkII N-SH3<br>Supplementary Data Table 5C: Summary of binary and ternary affinity of PEAK3 tandem peptides with 14-3-3 and CrkII N-SH3 determined using ITC or SPR<br>ITC data for binary and ternary binding experiments
Supplementary Data 6	Supplementary Data Table 6: SPR-determined binding affinity of full length or N-terminally truncated PEAK proteins with full length interactors or sub-domains<br>SPR sensorgrams and fitted steady state data
