# Supplementary material for: Structural mapping of PEAK pseudokinase interactions identifies 14-3-3 as a molecular switch for PEAK3 signaling
Source: Nat Commun. 2023 Jun 19;14:3542. doi: 10.1038/s41467-023-38869-9 (PMC10279719; doi:10.1038/s41467-023-38869-9)
Supplement: Supplementary file 5 — Reporting Summary [file 41467_2023_38869_MOESM5_ESM.pdf]

nature portfolio

Corresponding author(s): NCOMMS-22-34485A/LUCET

Last updated by author(s): May 5, 2023

Reporting Summary

Nature Portfolio wishes to improve the reproducibility of the work that we publish. This form provides structure for consistency and transparency in reporting. For further information on Nature Portfolio policies, see our Editorial Policies and the Editorial Policy Checklist.

Statistics

For all statistical analyses, confirm that the following items are present in the figure legend, table legend, main text, or Methods section.

n/a	Confirmed	
☐	☒	The exact sample size (*n*) for each experimental group/condition, given as a discrete number and unit of measurement
☐	☒	A statement on whether measurements were taken from distinct samples or whether the same sample was measured repeatedly
☐	☒	The statistical test(s) used AND whether they are one- or two-sided<br>*Only common tests should be described solely by name; describe more complex techniques in the Methods section.*
☒	☐	A description of all covariates tested
☒	☐	A description of any assumptions or corrections, such as tests of normality and adjustment for multiple comparisons
☐	☒	A full description of the statistical parameters including central tendency (e.g. means) or other basic estimates (e.g. regression coefficient) AND variation (e.g. standard deviation) or associated estimates of uncertainty (e.g. confidence intervals)
☐	☒	For null hypothesis testing, the test statistic (e.g. *F*, *t*, *r*) with confidence intervals, effect sizes, degrees of freedom and *P* value noted<br>*Give P values as exact values whenever suitable.*
☒	☐	For Bayesian analysis, information on the choice of priors and Markov chain Monte Carlo settings
☒	☐	For hierarchical and complex designs, identification of the appropriate level for tests and full reporting of outcomes
☒	☐	Estimates of effect sizes (e.g. Cohen's *d*, Pearson's *r*), indicating how they were calculated

Our web collection on statistics for biologists contains articles on many of the points above.

Software and code

Policy information about availability of computer code

Data collection	ASTRA (Wyatt Technology, v. 7.3.19), Biacore 8K Control Software (Cytiva, v. 3.0.12.15655), Biacore S200 Control Software (Cytiva, v. 1.1), MicroCal PEAQ-ITC Control Software (Malvern Panalytical, v. 1.41)<br><br>X-ray crystallography data collection was performed on the MX2 beamline at the Australian Synchrotron. The beamline/data collection were controlled using their standard in-house software (Blu-Ice, v. 5.0).<br><br>Mass spectrometry LC-MS/MS data collections were performed using the QExactive HF mass spectrometer (Thermo Scientific, Bremen, Germany) coupled online with an Ultimate 3000 RSLCnano system (Thermo Scientific, Bremen, Germany) and an HPLC Ultimate 3000, PAL, CTC autosampler coupled to a timsTOF Pro (Bruker) equipped with a CaptiveSpray source.<br><br>Imaging studies were performed using a Leica DFC9000 with Leica Application Suite X software (version: 3.7.5.24914)
Data analysis	AIMLESS (v. 0.5.21), ASTRA (Wyatt Technology, v.7.3.19), Biacore Insight Evaluation Software (Cytiva, v. 3.0.12.15655), Biacore S200 Evaluation Software (Cytiva, v. 1.1), MicroCal PEAQ-ITC Analysis software (v. 1.41), Byonic (Protein Metrics, v. 3.1.0), COOT (v. 0.9.8.1), Fiji (ImageJ) software (v. 2.0.0), PHASER (v. 2.8.3), PHENIX (v. 1.20.1-4487), XSCALE (v. 20161205), XDS (v. 20161205), Pymol (Schrodinger, v. 2.5.4), ChimeraX (UCSF, v. 1.5), GraphPad Prism (v.9.5.1), InDesign (Adobe, v.18.2.1), Illustrator (Adobe, v.27.5), Fiji (ImageJ) software (version: 2.0.0, Byonic (Protein Metrics, v. 3.1.0), MaxQuant (v. 1.6.17)

For manuscripts utilizing custom algorithms or software that are central to the research but not yet described in published literature, software must be made available to editors and reviewers. We strongly encourage code deposition in a community repository (e.g. GitHub). See the Nature Portfolio guidelines for submitting code & software for further information.

Data

Policy information about availability of data

All manuscripts must include a data availability statement. This statement should provide the following information, where applicable:

- Accession codes, unique identifiers, or web links for publicly available datasets
- A description of any restrictions on data availability
- For clinical datasets or third party data, please ensure that the statement adheres to our policy

Coordinates and structure factors for the X-ray crystal structures have been deposited in the PDB with accession codes 8DGO [http://doi.org/10.2210/pdb8DGO/pdb] (Grb2FL:PEAK SH2-pY peptide), 8DGP [http://doi.org/10.2210/pdb8DGP/pdb] (14-3-3ε:PEAK3tandem-pS69), 8DGM [http://doi.org/10.2210/pdb8DGM/pdb] (14-3-3ε:PEAK1tandem-pT1165) and 8DGN [http://doi.org/10.2210/pdb8DGN/pdb] (14-3-3ε: PEAK2tandem-pS826); and existing PDB structures 1GRI [http://doi.org/10.2210/pdb1GRI/pdb] (Grb2FL), 5IH2 [http://doi.org/10.2210/pdb5IH2/pdb] (CrkII-NSH3:Abl-758 peptide) and 2BR9 [http://doi.org/10.2210/pdb2BR9/pdb] (14-3-3ε). Coordinates for AlphaFold2 (AF2) models are available from the AlphaFold Protein Structure Database Q6ZS72 [http://http://alphafold.ebi.ac.uk/entry/Q6ZS72] (human PEAK3) and Q9H792 [http://alphafold.ebi.ac.uk/entry/Q9H792] (human PEAK1). Coordinates for the human PEAK3(SHED-PsK) dimer prepared from AlphaFold2 multimer modelling are provided in Source Data.

Research involving human participants, their data, or biological material

Policy information about studies with human participants or human data. See also policy information about sex, gender (identity/presentation), and sexual orientation and race, ethnicity and racism.

Reporting on sex and gender	N/A
Reporting on race, ethnicity, or other socially relevant groupings	N/A
Population characteristics	N/A
Recruitment	N/A
Ethics oversight	N/A

Note that full information on the approval of the study protocol must also be provided in the manuscript.

Field-specific reporting

Please select the one below that is the best fit for your research. If you are not sure, read the appropriate sections before making your selection.

☒ Life sciences ☐ Behavioural & social sciences ☐ Ecological, evolutionary & environmental sciences

For a reference copy of the document with all sections, see nature.com/documents/nr-reporting-summary-flat.pdf

Life sciences study design

All studies must disclose on these points even when the disclosure is negative.

Sample size	Statistical methods were not used to determine sample sizes.<br>For quantitation purposes, in general, unless otherwise stated a sample size of at least N =2 or N = 3 (two or three independent/biological repeats) was performed to enable assessment of the reproducibility and robustness of performed experiments. Sample sizes were selected based on similar studies in the field.<br>For biophysical analyses, in some cases where equivalent measurements were obtained using a second orthogonal method (e.g. SPR and ITC), reduced sample sizes of N = 1 or N = 2 were used for the second method, as the orthogonal nature of these methods provided additional confidence in the reproducibility of the data.
Data exclusions	No data were excluded from analyses unless otherwise noted.<br>For the SPR analysis, in some cases at the highest peptide concentration sensorgrams exhibited some non-specific binding; these data points were typically excluded from fitting (shown as dotted line in sensorgrams in Supplementary Data).
Replication	To ensure reproducibility of experimental findings, biochemical/biophysical or cellular assays were repeated independently at least 2-3 times under equivalent conditions or using orthogonal techniques. Specific replicate information can be found in figure legends. For interaction studies, a range of protein boundaries (ie. peptides, individual domains and full-length proteins) and orthogonal biophysical techniques were used to obtain binding data (e.g. ITC, SPR, SEC), to ensure reproducibility and specificity to the to the regions under study.<br>We confirm that all attempts at replication were successful.
Randomization	No experimental group allocation was carried out and so no randomization was performed.
Blinding	No experimental group allocation was carried out and so no blinding was performed.

Reporting for specific materials, systems and methods

We require information from authors about some types of materials, experimental systems and methods used in many studies. Here, indicate whether each material, system or method listed is relevant to your study. If you are not sure if a list item applies to your research, read the appropriate section before selecting a response.

Materials & experimental systems

n/a	Involved in the study
☐	☒ Antibodies
☐	☒ Eukaryotic cell lines
☒	☐ Palaeontology and archaeology
☒	☐ Animals and other organisms
☒	☐ Clinical data
☒	☐ Dual use research of concern
☒	☐ Plants

Methods

n/a	Involved in the study
☒	☐ ChIP-seq
☒	☐ Flow cytometry
☒	☐ MRI-based neuroimaging

Antibodies

Antibodies used

For cell based studies, the following antibodies were obtained commercially: anti-HA (Cell Signaling Technology, catalog no. 3724), anti-Flag (Sigma-Aldrich, catalog no. F1804), anti-14–3-3 (Santa Cruz Biotechnology, catalog no. sc-1657), anti-Grb2 (Cell Signaling Technology, catalog no. 3972S), anti-CrkII (Santa Cruz Biotechnology, catalog no. sc-289), p-Tyr (Cell Signaling Technology, catalog no. 8954S), b-actin (Santa Cruz Biotechnology, catalog no. sc-69879).

The following dilutions were used: 1:2000 for anti-HA and anti-Flag; 1:1000 for anti-14–3-3, anti-CrkII, anti-Grb2, anti-Tyr; 1:5000 for anti-actin antibodies.

Validation

All the used antibodies are commercially available and have been validated by the manufacturer to be used in that species/application and this information is provided on their websites and/or antibody datasheets. We confirm that the antibodies used are fit for purpose.

anti-HA (Cell Signaling Technology, catalog no. 3724).
See specific information from manufacturer: https://www.cellsignal.com/products/primary-antibodies/ha-tag-c29f4-rabbit-mab/3724

anti-Flag (Sigma-Aldrich, catalog no. F1804).
See specific details from manufacturer: https://www.sigmaaldrich.com/AU/en/product/sigma/f1804

anti-14–3-3 (Santa Cruz Biotechnology, catalog no. sc-1657).
See specific information from manufacturer: https://www.scbt.com/p/14-3-3-zeta-antibody-g-2

anti-Grb2 (Cell Signaling Technology, catalog no. 3972S).
See specific information from manufacturer: https://www.thermofisher.com/antibody/primary/target/grb2

anti-CrkII (Santa Cruz Biotechnology, catalog no. sc-289).
See specific information from manufacturer: https://www.scbt.com/p/crk-ii-antibody-c-18

p-Tyr (Cell Signaling Technology, catalog no. 8954S).
See specific information from manufacturer: https://www.cellsignal.com/products/primary-antibodies/phospho-tyrosine-p-tyr-1000-multimab-rabbit-mab-mix/8954

b-actin (Santa Cruz Biotechnology, catalog no. sc-69879).
See specific information from manufacturer: https://www.scbt.com/p/actin-antibody-h-6

Eukaryotic cell lines

Policy information about cell lines and Sex and Gender in Research

Cell line source(s)

HEK 293 cells were obtained from ATCC (CRL-1573).

The MCF-10A cell line stably expressing the murine ecotropic receptor (MCF-10A EcoR) was a gift from D. Lynch and J. Brugge, Harvard Medical School.

Authentication

HEK 293 were authenticated by ATCC as described on the ATCC website: https://www.atcc.org/products/crl-1573.
Both HEK 293 and MCF-10A EcoR cells were also authenticated by genetic polymorphism analysis.

Mycoplasma contamination	Both cell lines were routinely checked for mycoplasma contamination by PCR.
Commonly misidentified lines (See ICLAC register)	*Name any commonly misidentified cell lines used in the study and provide a rationale for their use.*
