# Supplementary material for: Structural mapping of PEAK pseudokinase interactions identifies 14-3-3 as a molecular switch for PEAK3 signaling
Source: Nat Commun. 2023 Jun 19;14:3542. doi: 10.1038/s41467-023-38869-9 (PMC10279719; doi:10.1038/s41467-023-38869-9)
Supplement: Supplementary file 6 — Source Data [file 41467_2023_38869_MOESM6_ESM.zip › Roy-NatComms_Source-Data.pdf]

Source data

Structural mapping of PEAK pseudokinase interactions identifies 14-3-3 as a molecular switch for PEAK3 signaling. Roy, M.J. *et al.*

Source data to Fig. 2: MS/MS analysis of tryptic peptides following *in vitro* kinase assay of PEAK3-FL and PEAK1^{IDR1} purified from insect cells. a, (i) PEAK3 control sample is not phosphorylated at Y24 (SH2 motif) in the absence of Src or Abl. (ii) PEAK3 is phosphorylated at Y24 (SH2 motif) upon incubation with Src. (iii) PEAK3 is not phosphorylated at Y24 (SH2 motif) upon incubation with Abl. **b,** (i) PEAK1^{IDR1} control sample is not phosphorylated at Y1107 (SH2 motif) in the absence of Src or Abl. (ii) PEAK1^{IDR1} is phosphorylated at Y1107 (SH2 motif) upon incubation with Src. (iii) PEAK1^{IDR1} is not phosphorylated at Y1107 (SH2 motif) upon incubation with Abl. **c,** Positive controls demonstrating Abl and Src *in vitro* kinase activity towards CrkII^{FL}. (i) CrkII^{FL} control sample is not phosphorylated at Y221 in the absence of Src or Abl. (ii) CrkII^{FL} is phosphorylated at Y221 (a known SFK site) upon incubation with Src. (iii) CrkII^{FL} is phosphorylated at Y221 (a known SFK site) upon incubation with Abl.

a. *In vitro* kinase assay of recombinant PEAK3^{FL}

(i) PEAK3 control sample is not phosphorylated at Y24 (SH2 motif) in the absence of Src or Abl.

PTWSTQTYSNLGQIR

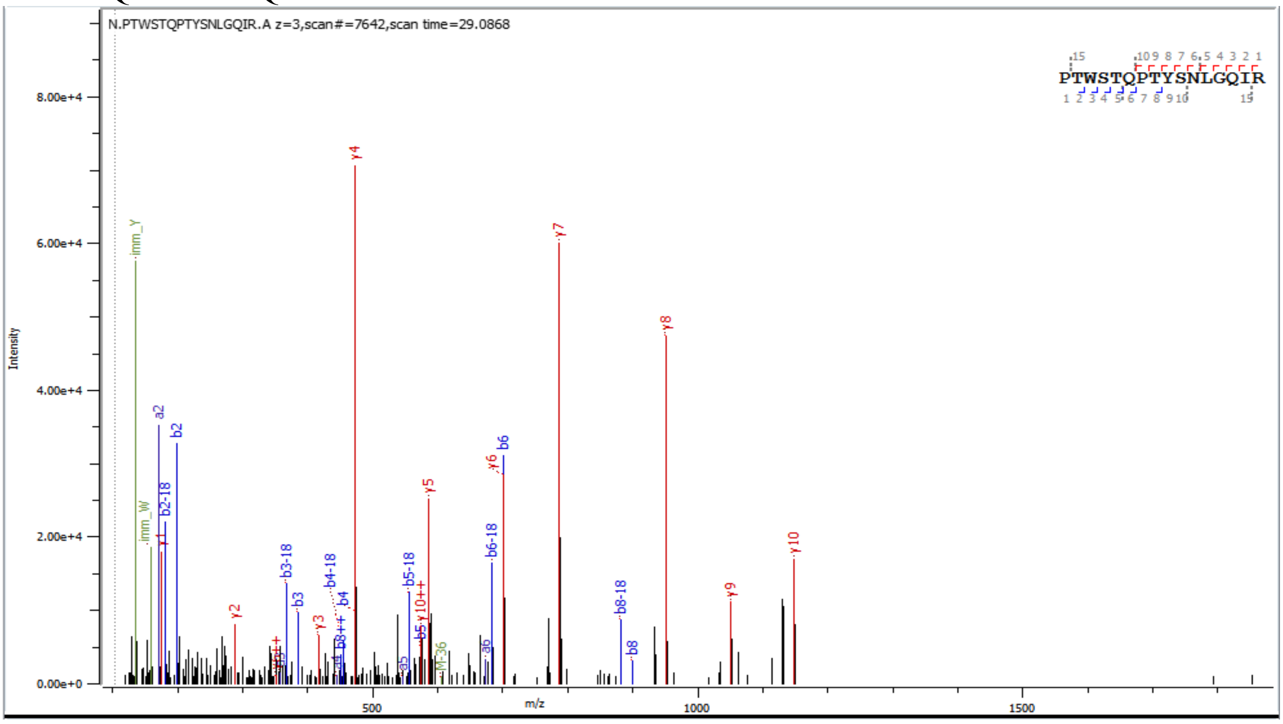

(ii) PEAK3 is phosphorylated at Y24 (SH2 motif) upon incubation with <u>Src</u>

PTWSTQPTY$_{p24}$SNLGQIR

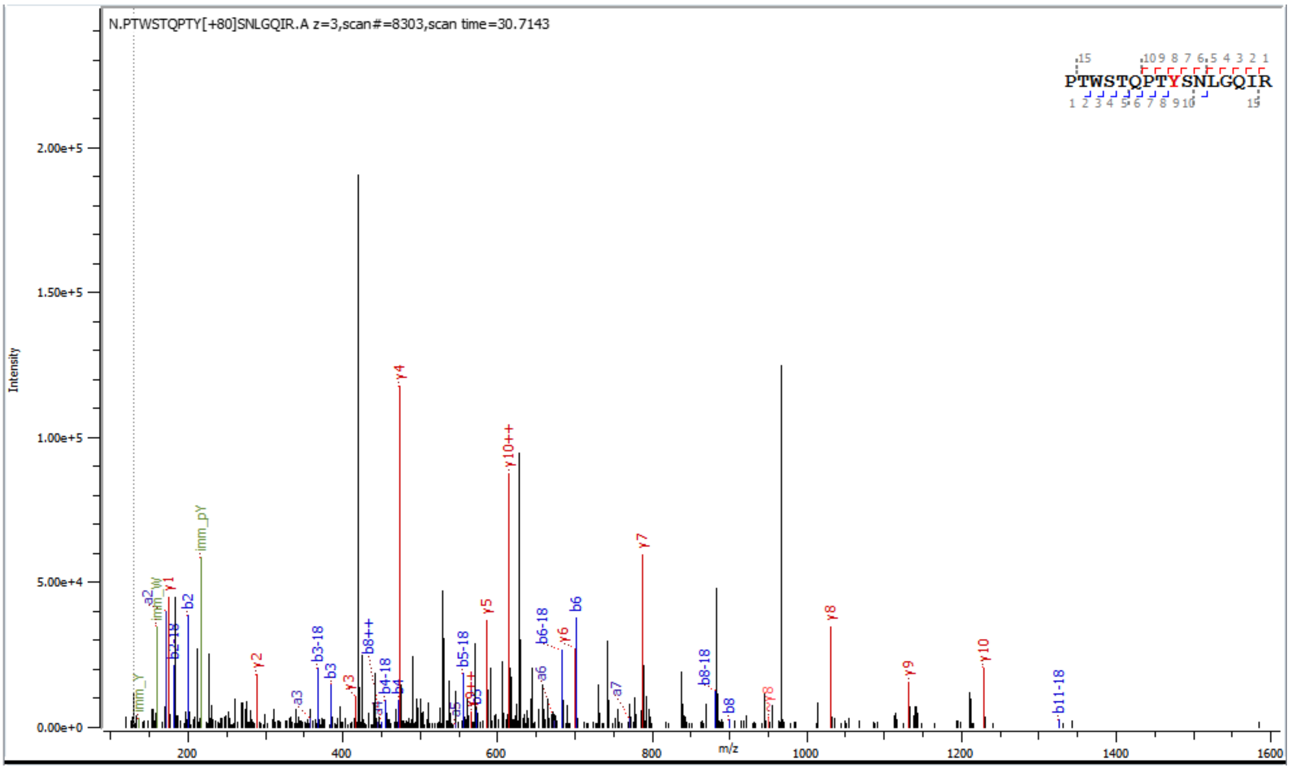

(iii) PEAK3 is <u>not</u> phosphorylated at Y24 (SH2 motif) upon incubation with <u>Abl.</u>

PTWSTQPTYSNLGQIR

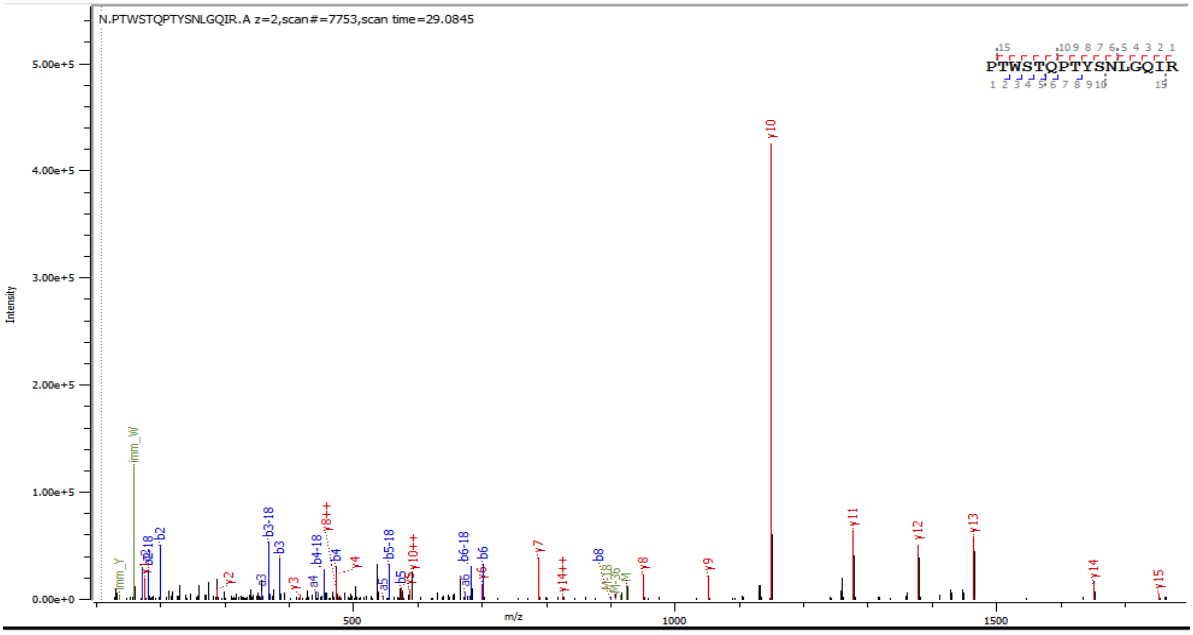

b. *In vitro* kinase assay - PEAK1^{IDR1} (*Sf*21)

(i) PEAK1^{IDR1} control sample is not phosphorylated at Y1107 (SH2 motif) in the absence of Src or Abl

EDGKEDISDPMDPNPCSATYSNLGQSR

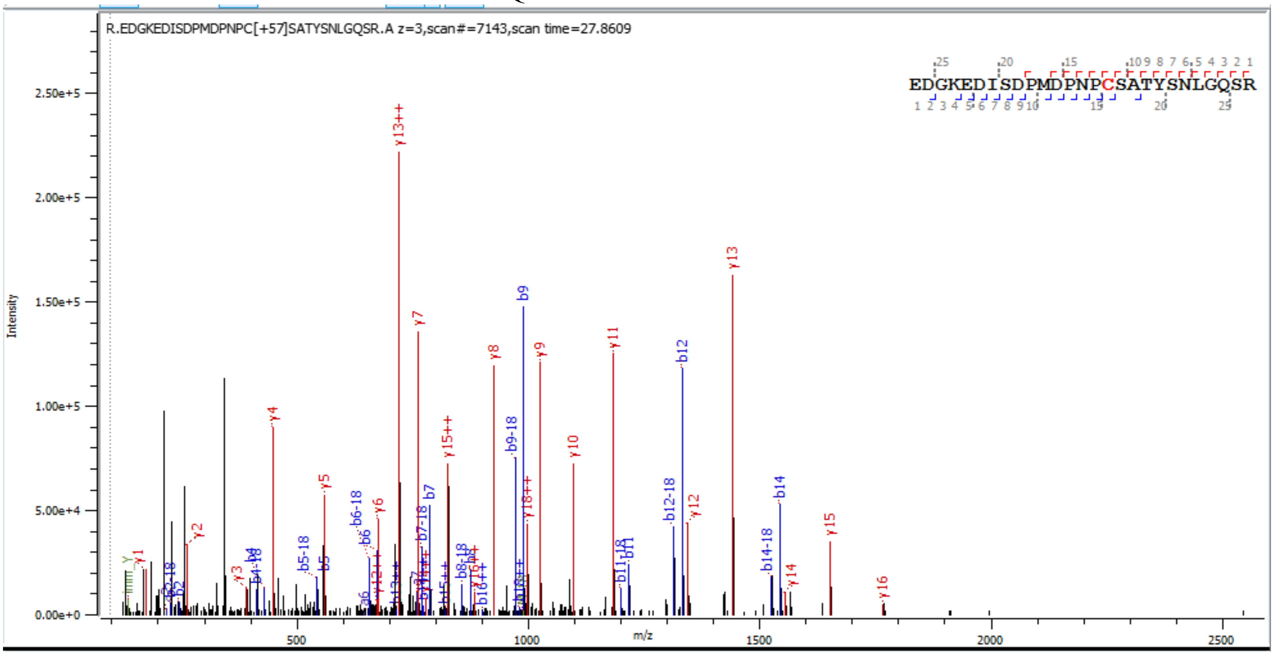

(ii) PEAK1^{IDR1} is phosphorylated at Y1107 (SH2 motif) upon incubation with Src

EDGKEDISDPMDPNPCSATY$_{p1107}$SNLGQSR

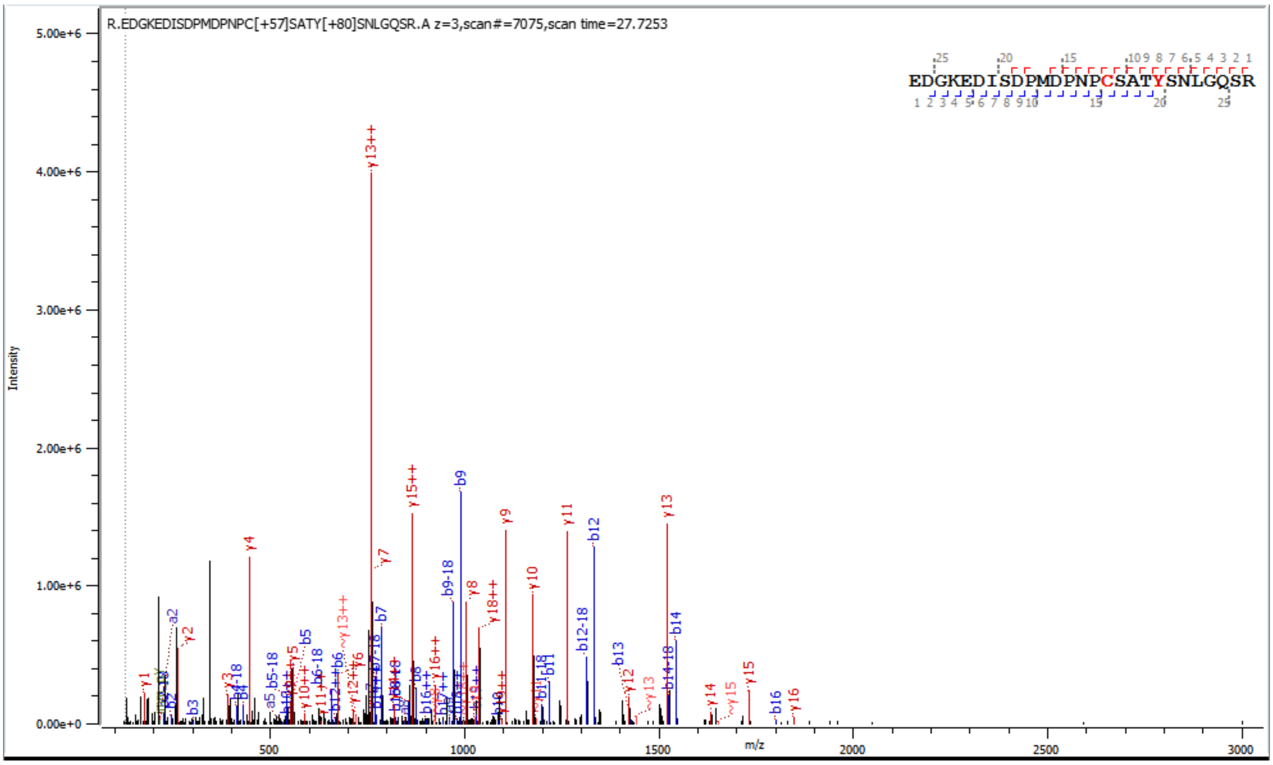

(iii) PEAK1^{IDR1} is not phosphorylated at Y1107 (SH2 motif) upon incubation with <u>Abl.</u>

SATYSNLGQSR

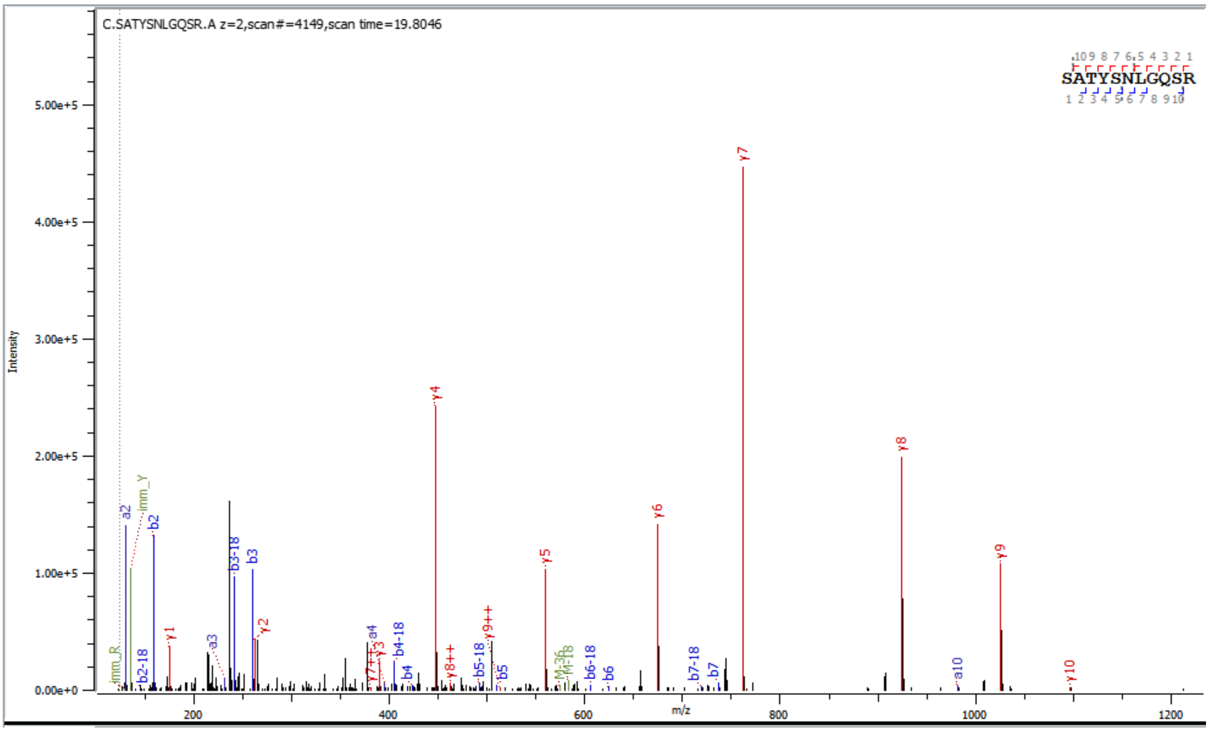

c. <u>*In vitro* kinase assay – CrkII^{FL} monomer (*E.coli*) – positive control</u>

(i) CrkII^{FL} control sample is not phosphorylated at Y221 in the absence of Src or Abl

YRPASASVSALIGGNQEGSHPQPLGGPEPGPYAQPSVNTPLPNLQNGPIYAR

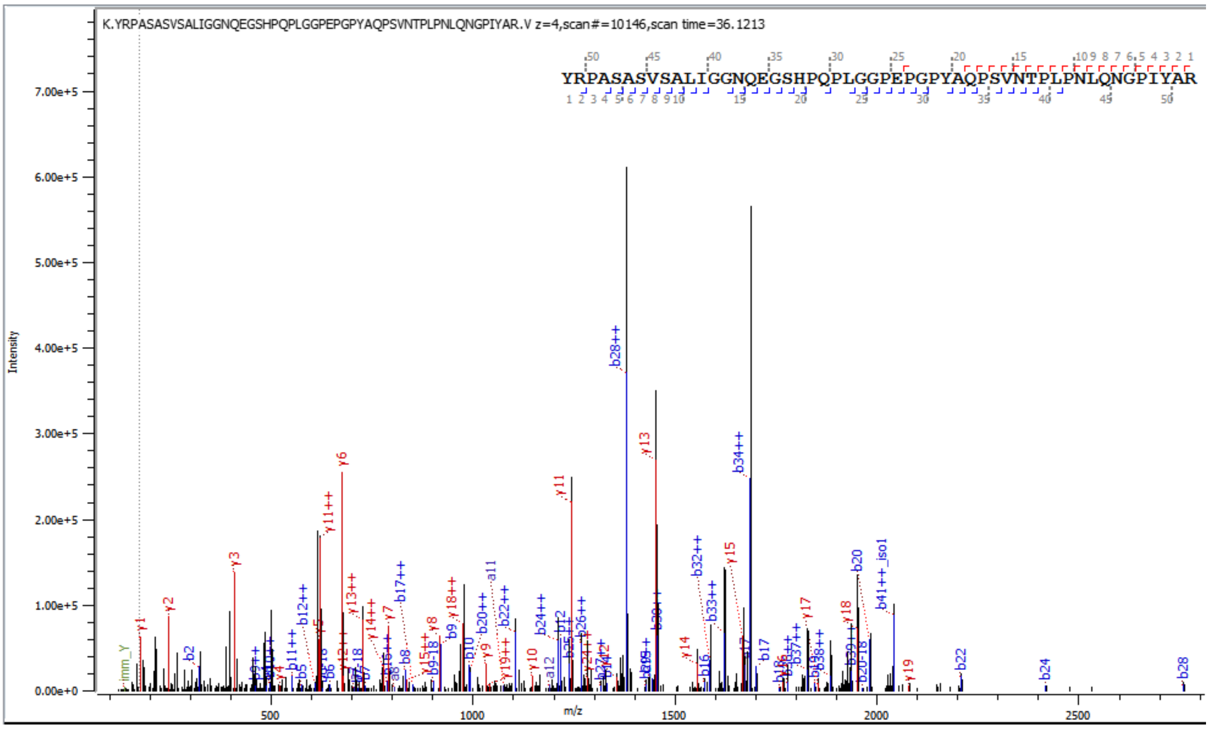

(ii) CrkII^FL is phosphorylated at Y221 (a known SFK site) upon incubation with <u>Src</u>

YRPASASVSALIGGNQEGSHPQPLGGPEPGPY$_{p221}$AQPSVNTPLPN

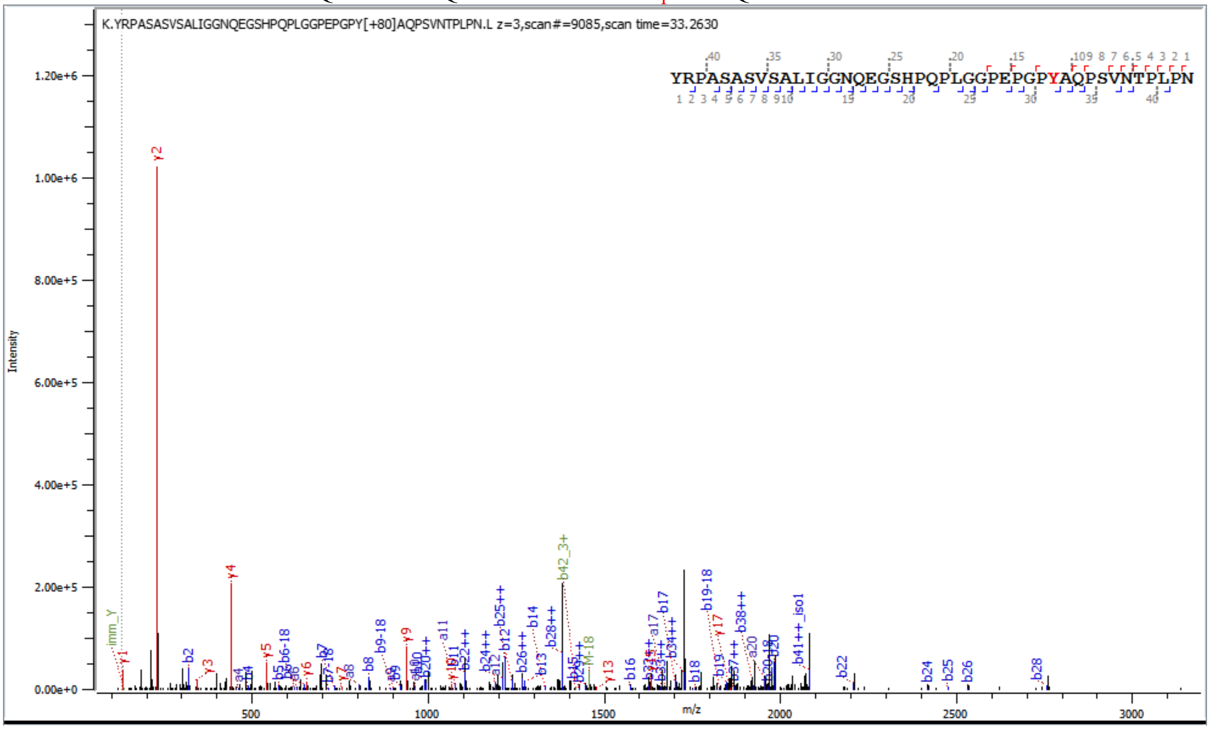

(iii) CrkII^FL is phosphorylated at Y221 (a known SFK site) upon incubation with <u>Abl</u>

YRPASASVSALIGGNQEGSHPQPLGGPEPGPY$_{p221}$AQPSVNTPLPNL

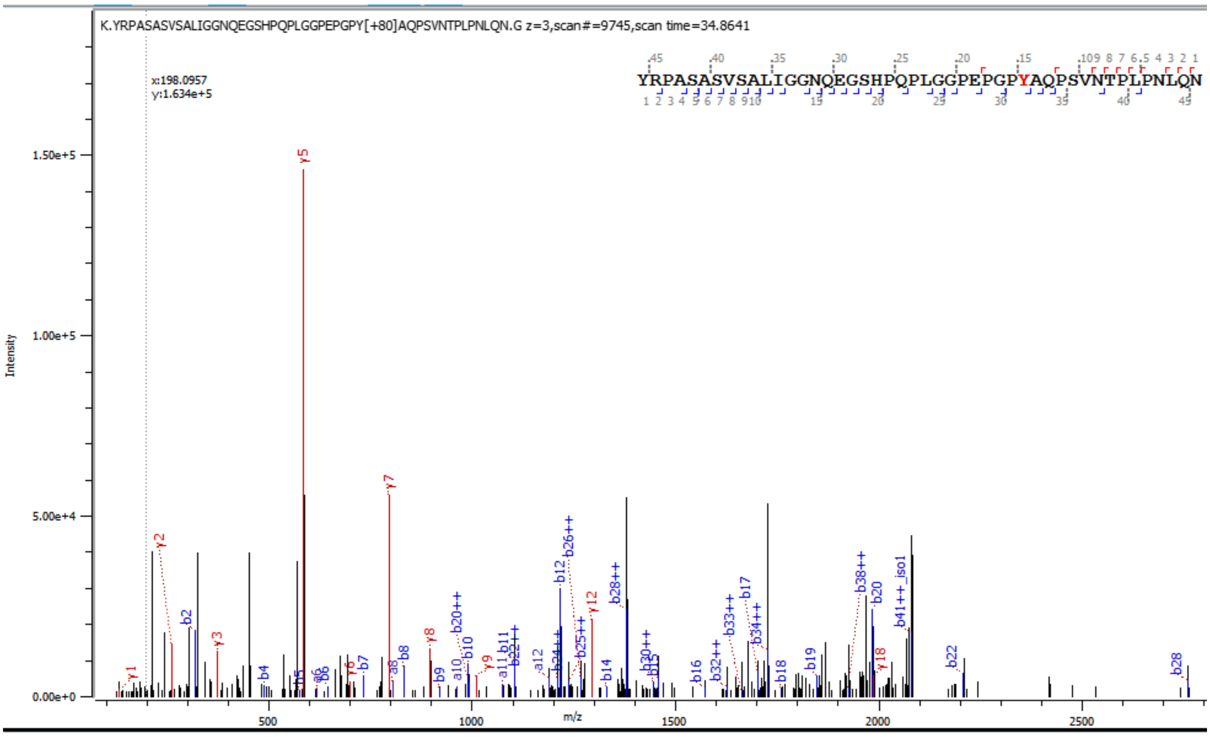

Source Data to Fig. 4a. (i), Tandem mass-spectrometry of tryptic peptides showing identification of 14-3-3ε and 14-3-3ζ isoforms as PEAK3 interactors. **(ii),** LC-MS/MS analysis of recombinant PEAK3 showing that the PEAK3 14-3-3 site is phosphorylated at S69 within the 14-3-3 motif (tandem site). **(iii),** LC-MS/MS analysis of recombinant insect cell expressed PEAK2^{IDR1} showing that it is phosphorylated at S826/S827 within the 14-3-3 motif (tandem site). **(iv),** LC-MS/MS analysis of recombinant insect cell expressed PEAK1^{IDR1} showing that it is not phosphorylated at the 14-3-3 motif (tandem site).

(i) Mass-directed tryptic proteomics showing identification of 14-3-3ε,ζ isoforms

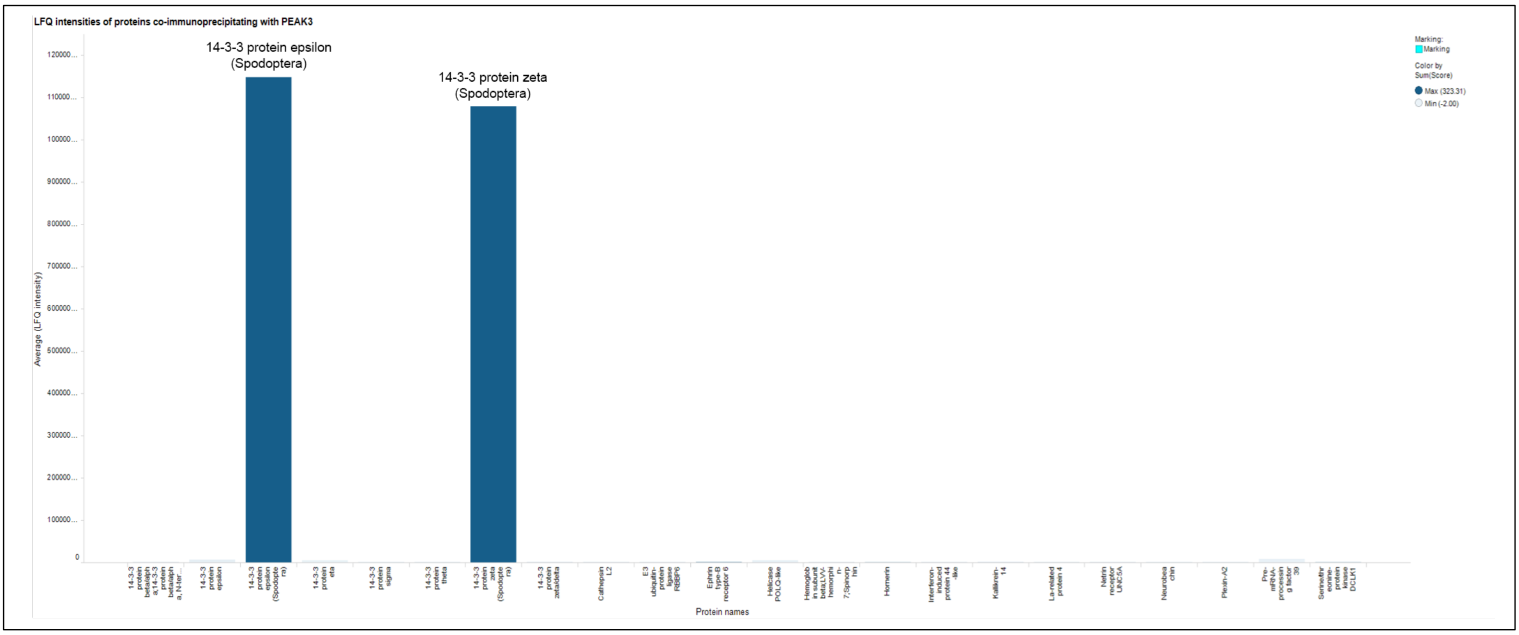

(ii) PEAK3 14-3-3 site is phosphorylated at S69 within the 14-3-3 motif (tandem site)

TQS$_{p69}$LPTRR

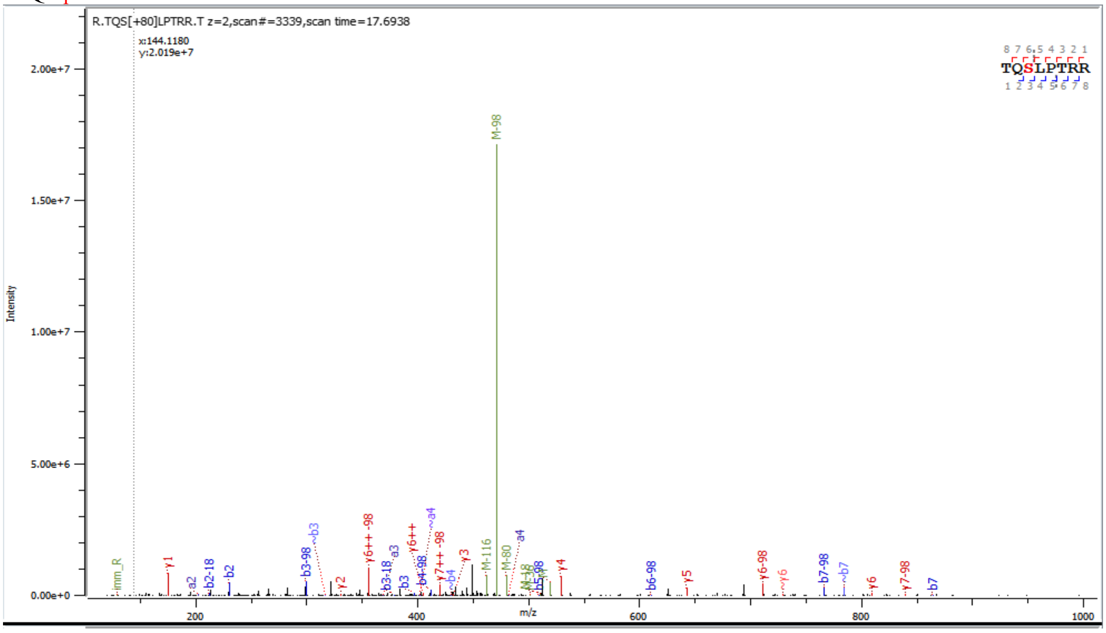

(iii) PEAK2$^{\text{IDR1}}$ is phosphorylated at S826/S827 within the 14-3-3 motif (tandem site)

AAS$_{\text{p826}}$SPDGFFWTQGSPK

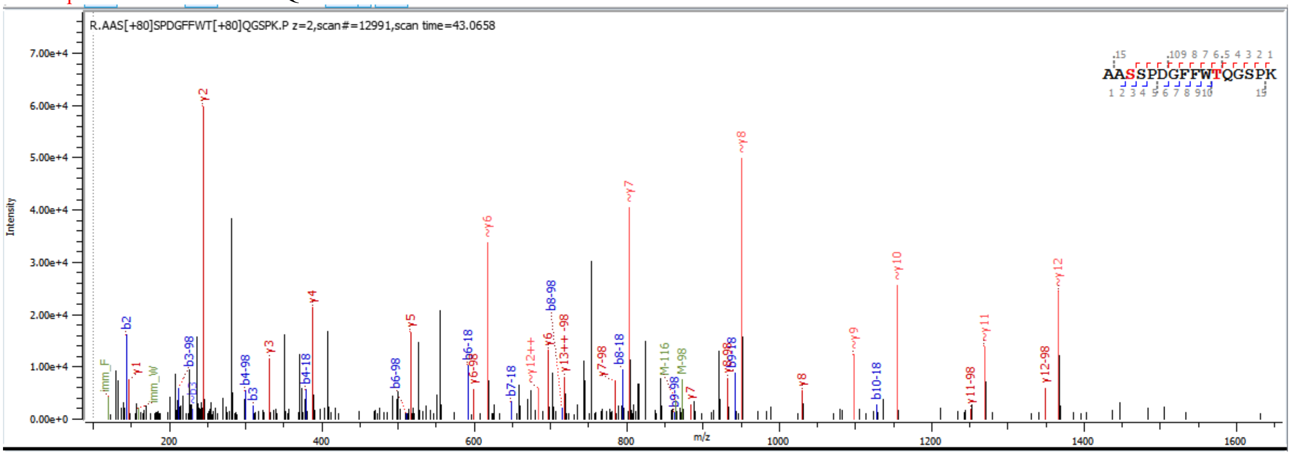

AASS$_{\text{p827}}$PDGFFWTQGSPK

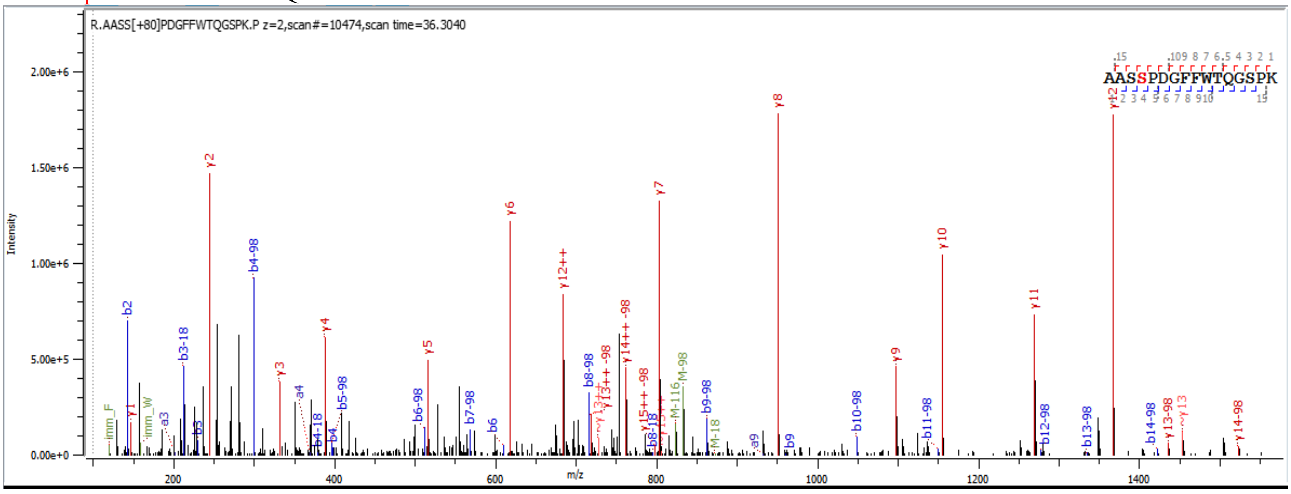

(iv) PEAK1$^{\text{IDR1}}$ is not phosphorylated within the 14-3-3 motif (tandem site)

ANTEPISKDLQK

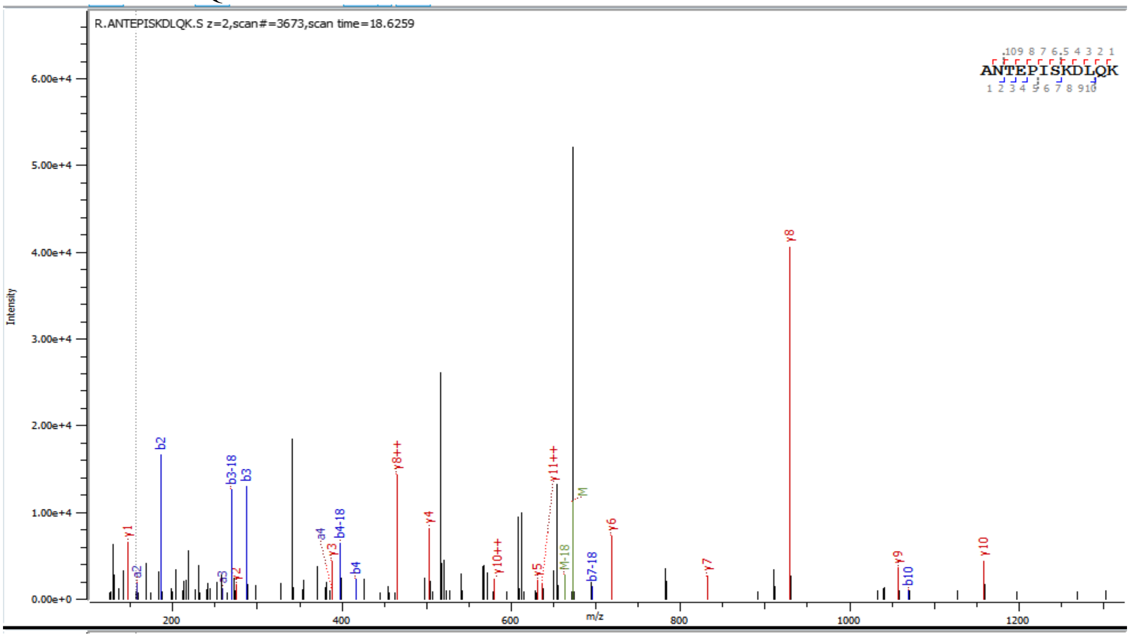

Source Data to Fig. 4b and Supplementary Fig. 4b. Uncropped gels

14-3-3 binding with PEAK3 S69A

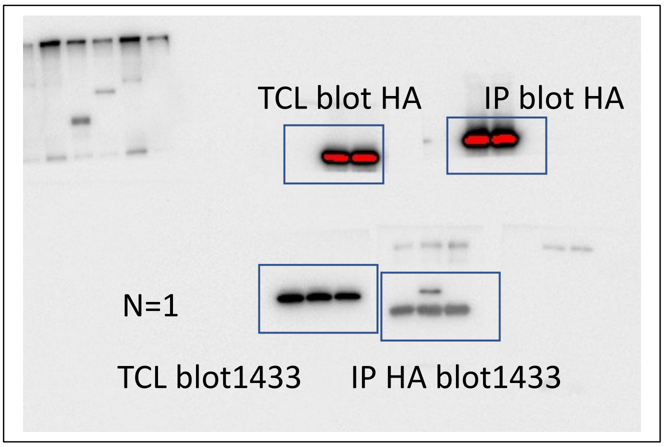

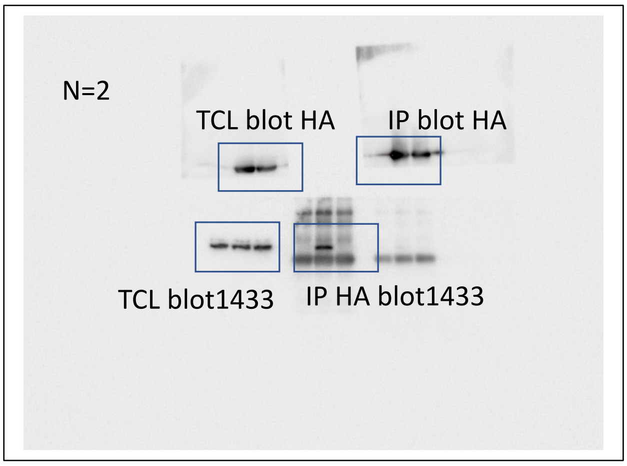

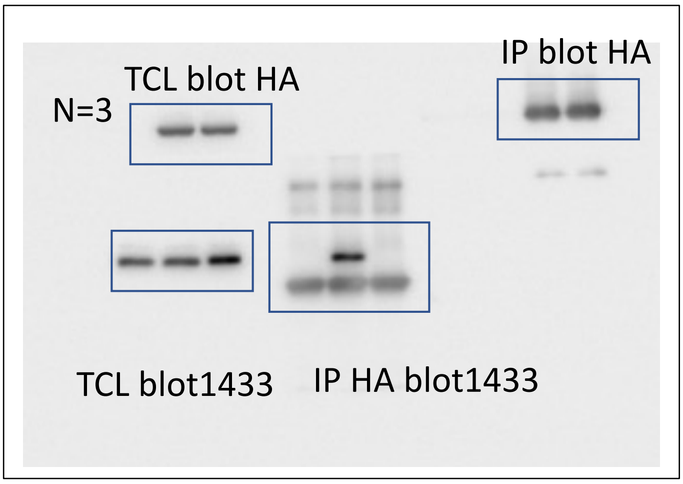

Source Data to Supplementary Fig. 4b. Uncropped gels

CrkII

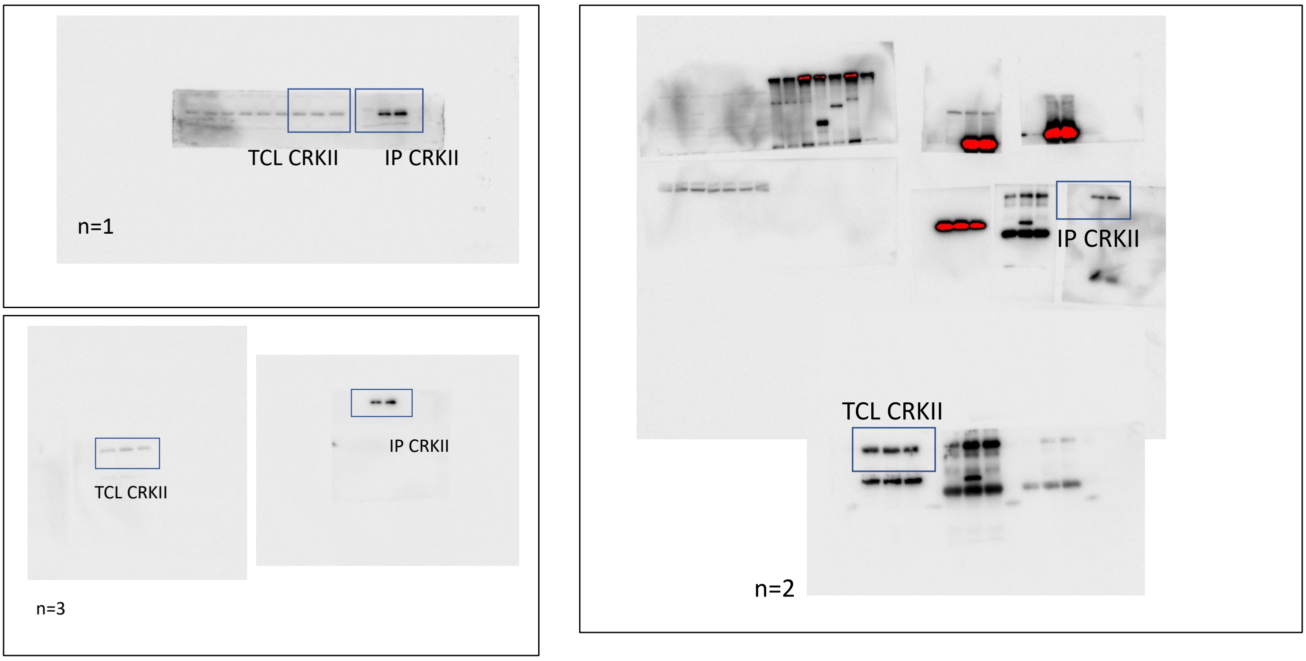

Grb2

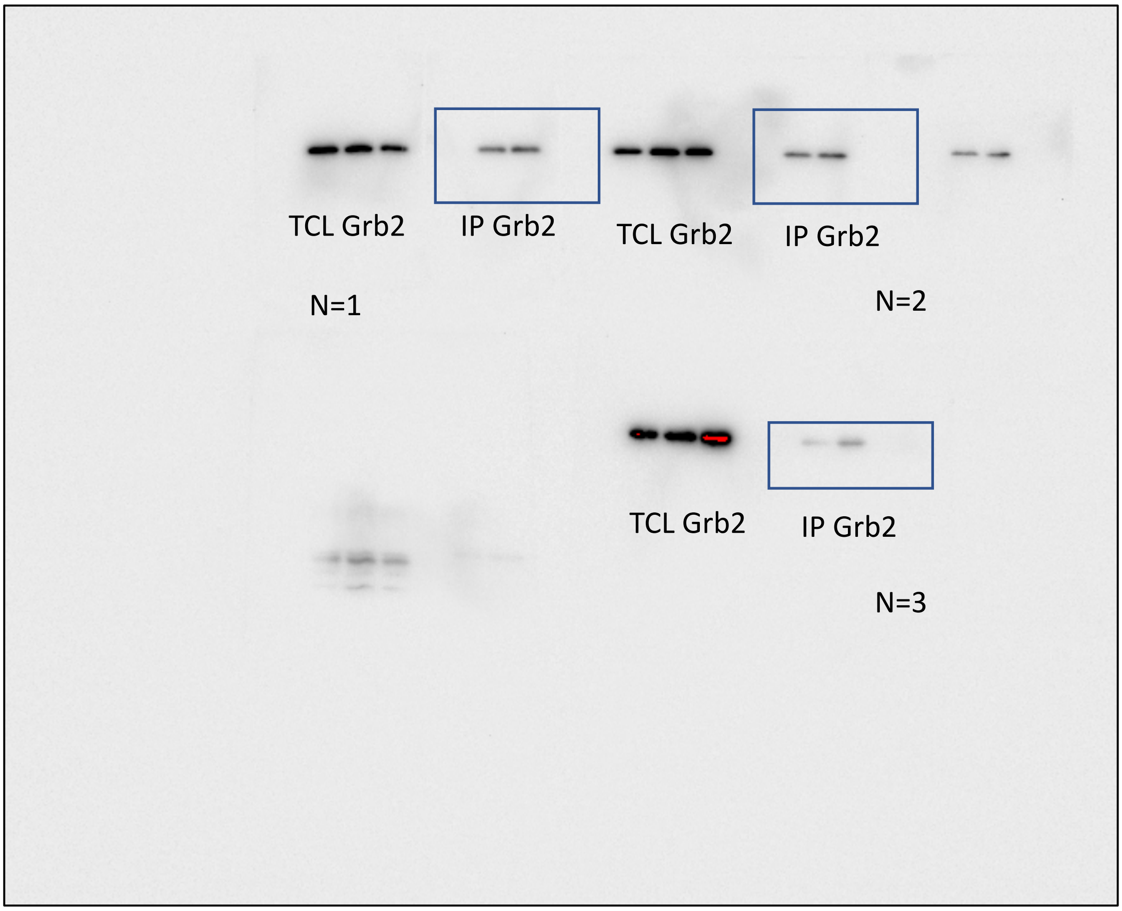

Source Data to Supplementary Fig. 4c. n=2 and n=3

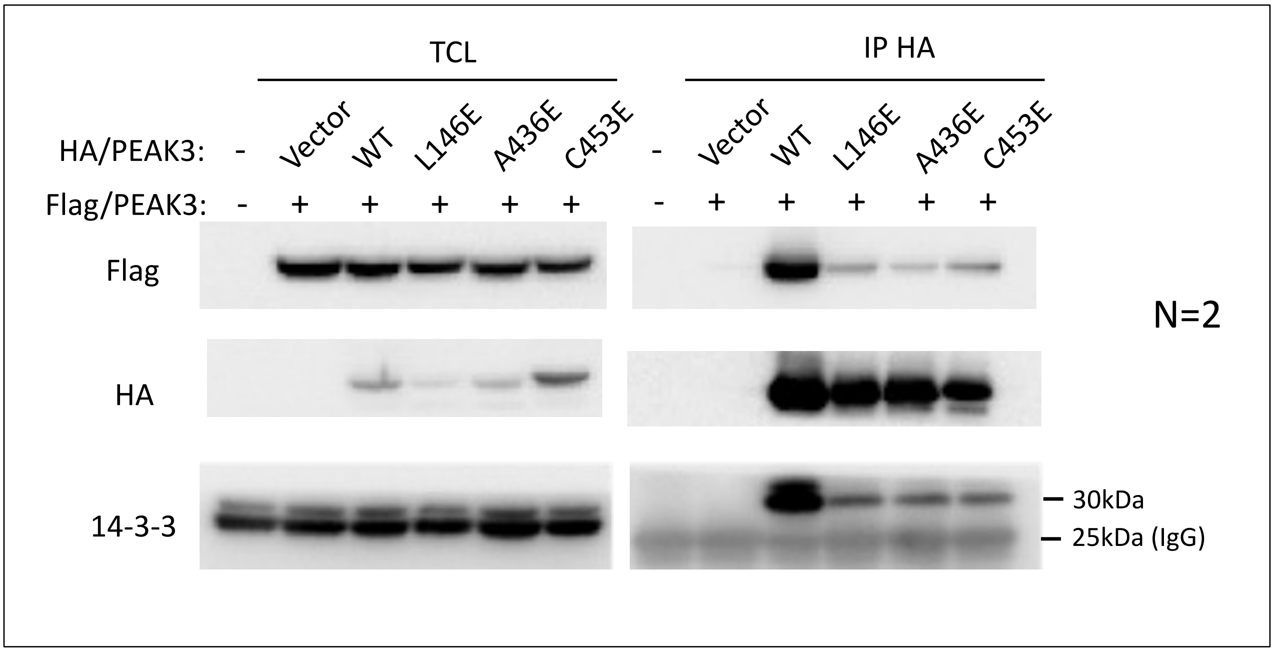

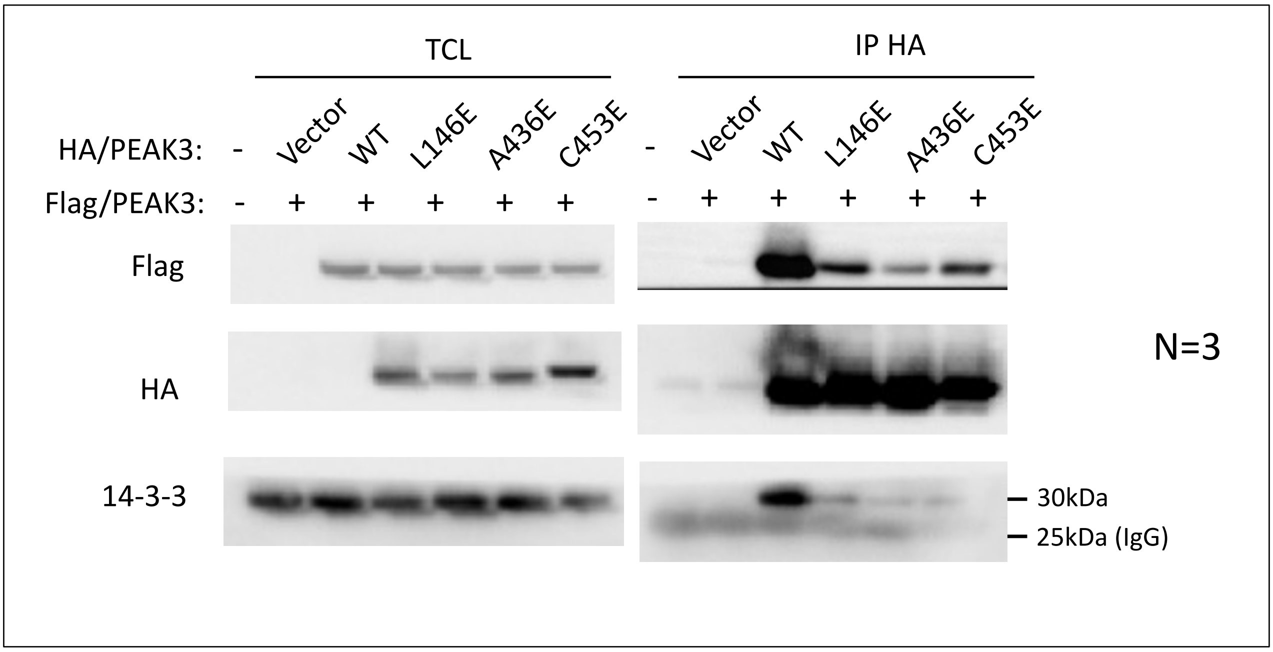

uncropped gels Supplementary Fig. 4c.

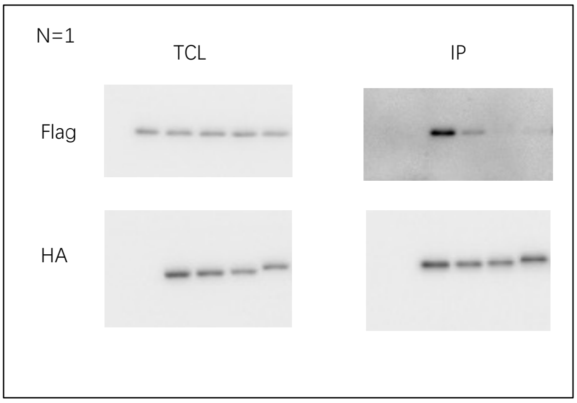

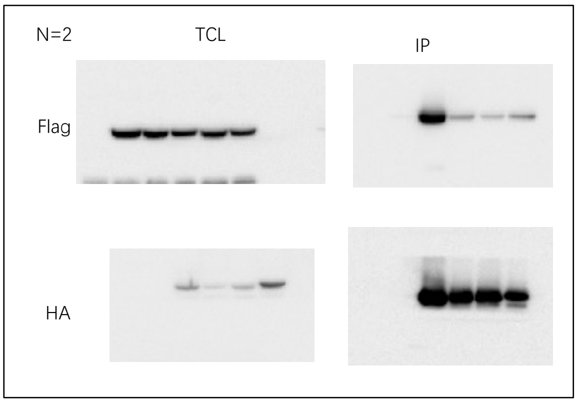

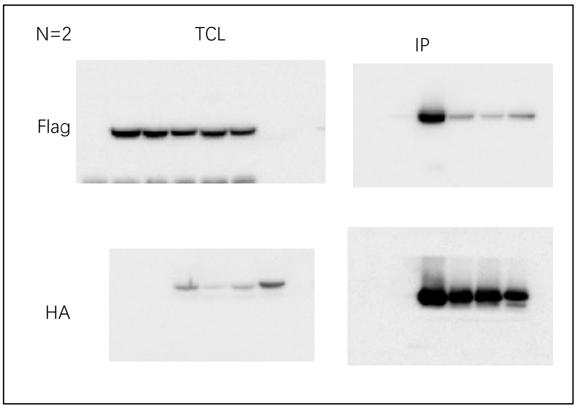

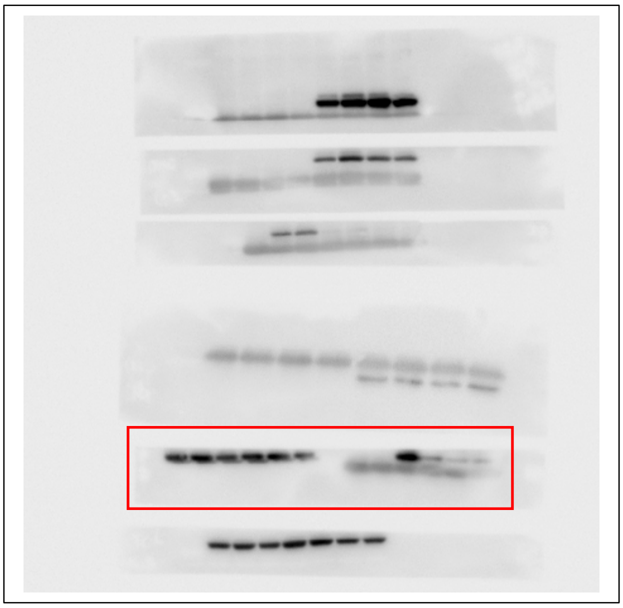

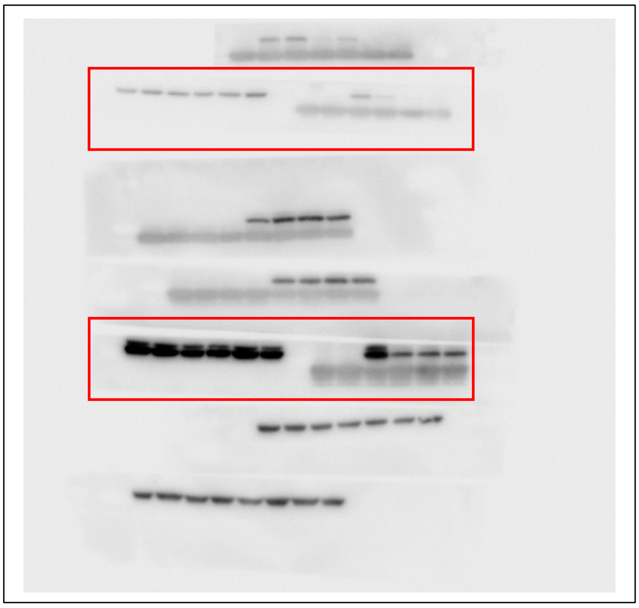

Raw data for Supplementary Figure 4b to generate densitometry graph data quantifying the mean level relative association of CrkII to HA-PEAK3^{FL} S69A relative to HA- PEAK3^{FL} WT ($p = 0.0073$), and of Grb2 to HA-PEAK3^{FL} S69A relative to HA- PEAK3^{FL} WT ($p = 0.0378$).

CrkII binding with PEAK3 S69A

	**WT**	**S69A**
n=1 CrkII blot	1	1.41
n=2 CrkII blot	1	1.37
n=3 CrkII blot	1	1.29

Grb2 binding with PEAK3 S69A

	**WT**	**S69A**
n=1 Grb2 blot	1	1.67
n=2 Grb2 blot	1	2
n=3 Grb2 blot	1	1.4

Source Data to Fig. 7a. Uncropped gels

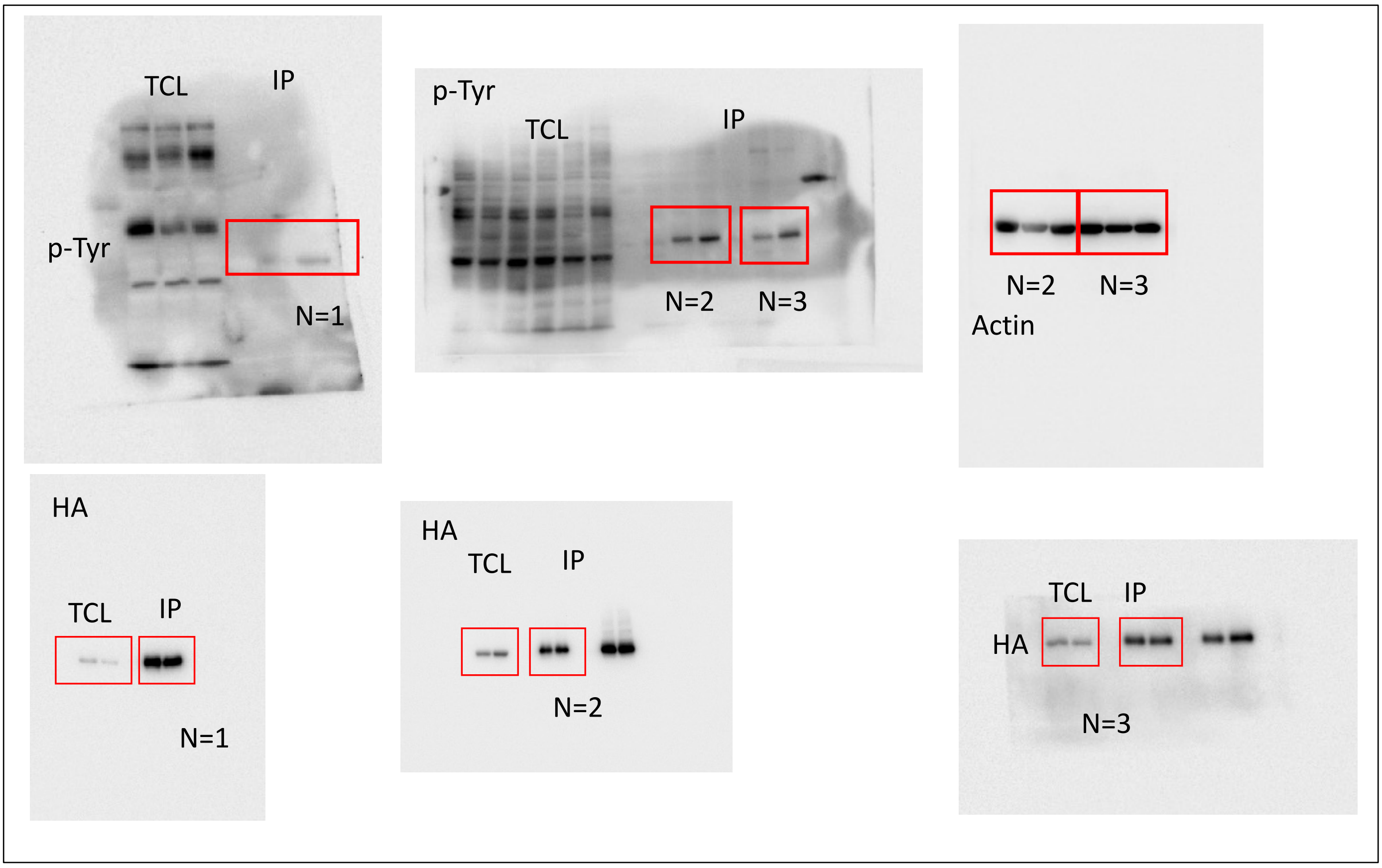

Raw data for Fig. 7a and 7b to generate histograms.

Fig7 a

	WT	S69A
n=1 relative p-Tyr blot	1	1.8
n=2 relative p-Tyr blot	1	2.2
n=3 relative p-Tyr blot	1	1.9

Fig7 b

	vector	WT	S69A
n=1 Average maximum distance (µm)	111	235	393
n=2 Average maximum distance (µm)	136	208	399
n=3 Average maximum distance (µm)	120	219	389
